# 2-Phenyl-4,4,5,5-tetramethylimidazoline-1-oxyl 3-oxide Radical (PTIO•) Trapping Activity and Mechanisms of 16 Phenolic Xanthones

**DOI:** 10.3390/molecules23071692

**Published:** 2018-07-11

**Authors:** Xican Li, Ban Chen, Xiaojun Zhao, Dongfeng Chen

**Affiliations:** 1 School of Chinese Herbal Medicine, Guangzhou University of Chinese Medicine, Waihuan East Road No. 232, Guangzhou Higher Education Mega Center, Guangzhou 510006, China; imchenban@foxmail.com (B.C.); zxj@gzucm.edu.cn (X.Z.); 2 Innovative Research & Development Laboratory of TCM, Guangzhou University of Chinese Medicine, Waihuan East Road No. 232, Guangzhou Higher Education Mega Center, Guangzhou 510006, China; 3 School of Basic Medical Science, Guangzhou University of Chinese Medicine, Waihuan East Road No. 232, Guangzhou Higher Education Mega Center, Guangzhou 510006, China; 4 The Research Center of Basic Integrative Medicine, Guangzhou University of Chinese Medicine, Waihuan East Road No. 232, Guangzhou Higher Education Mega Center, Guangzhou 510006, China

**Keywords:** xanthone, structure-activity relationship, antioxidant, *ortho*-di-OHs, *para*-di-OHs

## Abstract

This study used the 2-phenyl-4,4,5,5-tetramethylimidazoline-1-oxyl 3-oxide radical (PTIO•) trapping model to study the antioxidant activities of 16 natural xanthones in aqueous solution, including garcinone C, γ-mangostin, subelliptenone G, mangiferin, 1,6,7-trihydroxy-xanthone, 1,2,5-trihydroxyxanthone, 1,5,6-trihydroxyxanthone, norathyriol, 1,3,5,6-tetrahydroxy-xanthone, isojacareubin, 1,3,5,8-tetrahydroxyxanthone, isomangiferin, 2-hydroxyxanthone, 7-*O*-methylmangiferin, neomangiferin, and lancerin. It was observed that most of the 16 xanthones could scavenge the PTIO• radical in a dose-dependent manner at pH 4.5 and 7.4. Among them, 12 xanthones of the *para*-di-OHs (or *ortho*-di-OHs) type always exhibited lower half maximal inhibitory concentration (IC_50_) values than those not of the *para*-di-OHs (or *ortho*-di-OHs) type. Ultra-performance liquid chromatography coupled with electrospray ionization quadrupole time-of-flight tandem mass spectrometry (UPLC-ESI-Q-TOF-MS/MS) analysis revealed that most of these xanthones gave xanthone-xanthone dimers after incubation with PTIO•, except for neomangiferin. Based on these data, we concluded that the antioxidant activity of phenolic xanthone may be mediated by electron-transfer (ET) *plus* H^+^-transfer mechanisms. Through these mechanisms, some xanthones can further dimerize unless they bear huge substituents with steric hindrance. Four substituent types (i.e., *para*-di-OHs, 5,6-di-OHs, 6,7-di-OHs, and 7,8-di-OHs) dominate the antioxidant activity of phenolic xanthones, while other substituents (including isoprenyl and 3-hydroxy-3-methylbutyl substituents) play a minor role as long as they do not break the above four types.

## 1. Introduction

**Figure 1:**
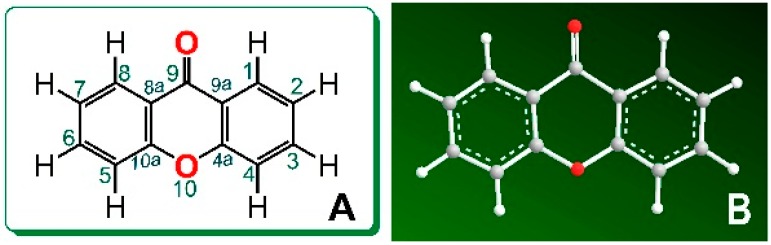
The structure (**A**) and preferential conformation (**B**) of the xanthone scaffold.

Natural xanthones can be isolated from edible plants, medicinal plants (including Chinese herbal medicines [1]), and marine-derived fungi (e.g., *Talaromyces islandicus* EN-501 [2]). Particularly, dozens of xanthones have been successfully isolated from the tropical fruits mangosteen and mango, and xanthones have been considered as the bioactive constituents of these fruits [3,4,5,6]. Structural elucidation demonstrated that the xanthone scaffold is composed of two phenyl rings and one pyrone ring in the same plane (Figure 1). In this planar and symmetrical scaffold, the hydrogen atom (H) at the 1–8 position can be substituted by -OH to construct phenolic -OHs; thus, xanthones can be regarded as natural phenolics and serve as phenolic antioxidants [3,4,7].

Phenolic antioxidants were reported to play an important role in disease prevention [5]. For example, mangosteen fruit was recently suggested to have an anti-tumor effect towards hepatic carcinoma [8]. Indeed, anti-tumor activity is closely associated with antioxidant activity. This is because cell carcinogenesis results—to a great extent—from reactive oxygen species (ROS)-induced oxidative damage [9]. Phenolic xanthones and other phenolics can effectively suppress the excessive ROS to prevent carcinogenesis. Nevertheless, a systematic investigation of the antioxidant activities and mechanisms of the xanthones family has not yet been reported.

**Figure 2:**
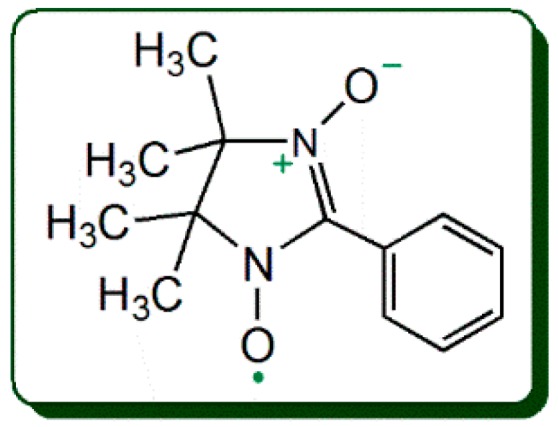
The structure of the 2-phenyl-4,4,5,5-tetramethylimidazoline-1-oxyl 3-oxide radical (PTIO•).

In the study, a 2-phenyl-4,4,5,5-tetramethylimidazoline-1-oxyl 3-oxide radical (PTIO•, Figure 2) trapping assay was introduced for the investigation. The PTIO•-trapping model has been newly developed by our team [10], and has at least two advantages over the common antioxidant assays, e.g., 1,1-diphenyl-2-picryl-hydrazl (DPPH•) scavenging assay and 2,2′-azino-bis(3-ethyl-benzothiazoline-6-sulfonic acid) (ABTS•^+^) scavenging assay. Like the •OH radical, the •O_2_- anion radical, and other ROS, the PTIO• radical is also an oxygen-centered radical (Figure 2); Like antioxidant action in cells, PTIO• trapping action is also fulfilled in aqueous media; aqueous media however is more biologically relevant [11,12]. Two common antioxidant assays (especially the DPPH•-scavenging assay), however, are based on a nitrogen-centered radical and performed in lipophilic media. Thus, the PTIO•-trapping assay can well characterize the ROS-scavenging action of an antioxidant and is more suitable to study the antioxidant activity of phenolic xanthones. To research their antioxidant mechanisms, the reaction products of xanthones with PTIO• were further determined using ultra-performance liquid chromatography coupled with electrospray ionization quadrupole time-of-flight tandem mass spectrometry (UPLC−ESI−Q−TOF−MS/MS) in the study.

Hypothetically, the antioxidant activities and mechanisms should arise from the effectiveness of some specific substituents in xanthones. As indicated in the literature [13,14], these substituents mainly refer to phenolic -OH, isoprenyl, cyclized-isoprenyl, methyl, and glycoside. Phenolic -OH, however, can be further classified into several types: *para*-di-OHs, 5,6-di-OHs, 6,7-di-OHs, 7,8-di-OHs, single phenolic -OH, and *meta*-di-OHs. The isoprenyl substituent is considered as a characteristic of the xanthones family, because about 50% members contain this substituent [13,14]. Whether and how these substituents affect the antioxidant activities of xanthones remain unknown until now, despite the fact that at least 300 phenolic xanthones have already been recorded in the literature [13,14].

**Figure 3:**
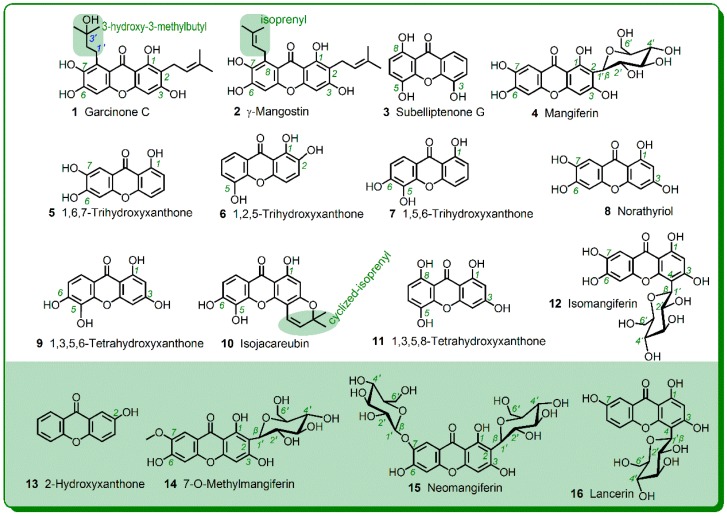
Structures of the 16 selected xanthones.

To address the above problems, 16 xanthones were randomly selected as references in this study (Figure 3). As seen in Figure 3, these references covered all the aforementioned substituents. Moreover, they account for about 6% of whole xanthones [13,14]. Thereby, the study is expected to provide new and characteristic information of phenolic xanthones.

## 2. Results and Discussion

### 2.1. Antioxidant Activity and Mechanism in PTIO•-Trapping Assay

As suggested by the cyclic voltammogram, PTIO•-trapping below pH 5.0 is an ET process [10]. In the present study, most of xanthones could scavenge the PTIO• radical in a dose-dependent manner at pH 4.5 (Supplementary Materials S1). This indicated that xanthones might possess ET potential during the antioxidant process. Furthermore, the PTIO•-trapping activities of these xanthones were also determined at physiological pH (7.4). PTIO•-trapping action at pH 7.4 may be involved in H^+^-transfer [10]. As shown in Supplementary Materials S1, the PTIO•-trapping percentages of the 16 xanthone references also increased in a concentration-dependent manner at pH 7.4, implying that the antioxidant activity of xanthone may also be involved in H^+^-transfer. In fact, at physiological pH, phenolic -OH with weak acidity may ionize to give rise to H^+^. For example, mangiferin has been demonstrated to be of pK_a1_ 6.52 ± 0.006 (25 °C) [15]. At pH 4.5, its weak acidity may be suppressed by the solution H^+^ ion, thus its H^+^-transfer has been reduced. Accordingly, it exhibited higher IC_50_ value at pH 4.5, compared with at pH 7.4 (Table 1).

**Figure 4:**
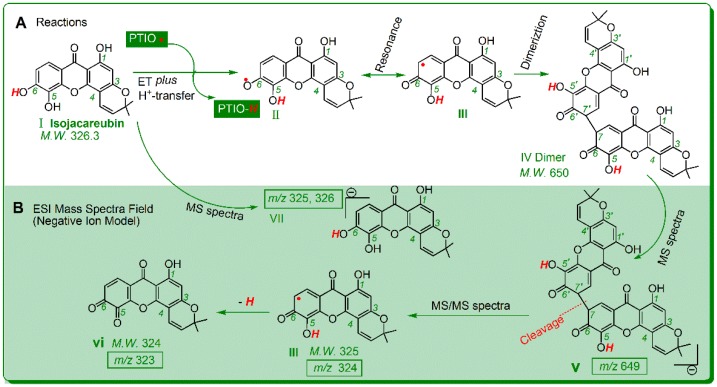
The possible reactions of isojacareubin with PTIO• (**A**), and the MS spectra elucidations (**B**) (The *m/z* value is expressed as an integer; precise *m/z* values are detailed in Supplementary Materials S18.).

To study the antioxidant mechanisms further, each of xanthones was incubated with the PTIO• radical and the reaction product was subsequently analyzed using UPLC−ESI−Q−TOF−MS/MS. The results in Table 1 revealed that most of these xanthones gave rise to a dimeric product. For example, after incubation with the PTIO• radical, isojacareubin yielded an isojacareubin-isojacareubin dimer, which displayed a primary MS peak (*m*/*z* 649) under negative ion model. This primary MS peak was further observed to cleave to produce *m*/*z* 323 and 324 (Table 1 and Supplementary Materials S18). Based on these MS spectral data and previous work [16], the dimerization reaction of isojacareubin can be proposed as Figure 4A; while the MS spectra elucidation is described in Figure 4B.

In the proposed reactions (Figure 4), the covalent bond between the 7- and 7′- positions has linked two isojacareubin radicals (III), to generate one isojacareubin-isojacareubin dimer (IV). The generation of a dimer can be considered as the consequence of ET *plus* H^+^-transfer (Figure 4). If there is no H^+^-transfer, there will be no peak of *m/z* 649 in the product. On the other hand, if there is only H^+^-transfer and no ET, there will be a phenoxy anion; two phenoxy anions however cannot combine with each other via covalency. Therefore, the evidence from UPLC−ESI−Q−TOF−MS/MS analysis can further verify the occurrence of ET *plus* H^+^-transfer.

It is worth mentioning that, (i) the dimerization reaction may be more complicated than that in Figure 4, and the covalent bond can also be linked to other positions [17,18,19]. Nevertheless, it is doubtless that the dimeric isojacareubin-isojacareubin is formed. (ii) The only xanthone that did not give a dimer product was neomangiferin (Table 1) bearing two sugar residues. This can be attributed to the fact that the two sugar residues have huge substituents and may hinder the radical adduct formation (RAF) potential. The RAF product however was supported by the earlier literatures where it was termed as nonradical product [11,20,21].

### 2.2. Antioxidant Role of Phenolic -OH: Evidence From PTIO•-Trapping Assay

Results from the colorimetric method revealed that there was a great difference in the H^+^-transfer (or ET) potentials among the 16 references (Table 1). At pH 4.5, the values of IC_50_ varied from 36.0 μM to 681.2 μM. Particularly, there was an evident gap in IC_50_ values at pH 4.5 between the former twelve xanthones (**1**–**12**, IC_50_ = 36.0–121.3 μM, pH = 4.5) and the latter four xanthones (**13**–**16**, IC_50_ = 284.2–681.2 μM, pH = 4.5, Table 1). The latter four xanthones contained neither *para*-di-OHs nor *ortho*-di-OHs. In this case, even with multiple phenolic -OHs, the xanthone still exhibited very low high IC_50_ values, e.g., neomangiferin (**15**). The data of neomangiferin clearly indicated that, *para*-di-OHs (or *ortho*-di-OHs) played a critical role in antioxidant activity at pH 4.5; and single phenolic -OHs and *meta*-di-OHs type played a negligible role. As seen in Table 1, both *para*-di-OHs and *ortho*-di-OHs xanthones are classified into the strong group based on the IC_50_ values at pH 4.5, implying that the role of *para*-di-OHs is roughly equivalent to that of *ortho*-di-OHs. A typical example was the pair of **9** vs. **11** at pH 4.5; A similar situation was also observed at pH 7.4. At pH 7.4, however, the PTIO•-trapping has been mentioned to be mediated by H^+^-transfer [10]. Thus, it can be inferred that *para*-di-OHs or *ortho*-di-OHs may similarly govern the ET *plus* H^+^-transfer potentials.

Further analysis suggested that *ortho*-di-OHs could be divided into 3 types, i.e., 5,6-di-OHs (e.g., **7**, **9**, and **10**), 6,7-di-OHs (e.g., **1**, **2**, **4**, **5**, **8**, and **12**), and 7,8-di-OHs (e.g., **6**). The fact that the IC_50_ values of 3 xanthones (1,6,7-trihydroxyxanthone (**5**), 1,2,5-trihydroxyxanthone (**6**), and 1,5,6-trihydroxyxanthone (**7**)) are not significantly different (*p* > 0.05) at pH 4.5 suggests that the *ortho*-di-OHs positions (5,6-position, 6,7- position, or 7,8-position) have not affected the antioxidant activity. This suggestion can partly explain the similarities of a pair of xanthones: norathyriol (**8**) and its isomer 1,3,5,6-tetrahydroxyxanthone (**9**). As shown in Table 1, in PTIO•-trapping colorimetric analysis, there is no significant difference (*p* > 0.05) in IC_50_ values at pH 4.5 between them. In PTIO•-trapping UPLC−ESI−Q−TOF−MS/MS analysis, norathyriol and its isomer produced a similar series of MS peaks (Table 1 and Supplementary Materials S18). Based on the MS spectra elucidation, it is presumed that these two isomers have undergone similar dimerization reactions (Supplementary Materials S19,20).

In short, during the PTIO•-trapping process, *meta*-di-OHs have a negligible effect on the occurrence of ET *plus* H^+^-transfer reactions, and each of the other four substituent types (*para*-di-OHs, 5,6-di-OHs, 6,7-di-OHs, and 7,8-di-OHs) can govern the antioxidant activity of phenolic xanthones. However, if any of the four types is broken, the antioxidant activity is remarkedly decreased. For example, when 6,7-di-OHs in mangiferin (**4**) were broken by a glucoside to form neomangiferin (**15**), the IC_50_ values were greatly increased (8.5-times for pH 4.5 and 5.6-times for pH 7.5, Table 1).

Our findings regarding the role of *ortho*-di-OHs was further supported by a number of reports, which usually referred to *ortho*-di-OHs as the catechol moiety [22,23,24,25,26]. The reason why the *ortho*-di-OHs play a critical role in antioxidant xanthones may be the fact that *ortho*-di-OHs can be oxidized to *ortho*-benzo quinone by free radicals [27,28]. For example, in the reaction of isojacareubin with PTIO•, 5,6-di-OHs is oxidized to 5,6-*ortho*-benzoquinone via ET *plus* H^+^-transfer mechanisms [29,30] (Figure 4). Similarly, *para*-di-OHs type could be hypothesized to transform into *para*-benzoquinone, in accordance with Figure 4. In fact, the *para*-di-OHs molecule itself has strong antioxidant activity. It is now clear that the stability of *para*-benzoquinone or *ortho*-benzoquinone can be responsible for the strong antioxidant activity of *para*-di-OHs (or *ortho*-di-OHs) type. Our experimental data and deduction denied the opinion of a minor role of *ortho*-di-OHs type (catechol moiety) [31].

It must be emphasized that (1) the antioxidant role of *para*-di-OHs type in natural phenolics has not been mentioned previously [32]. This may be attributed to the fact that *para*-di-OHs are hardly found in flavonoids [13,14,26,33] and other phenolics [13,14,19,22,23,24,25,26,27,28,29,30,31,34,35]. For instance, among thousands of flavonoids, only 5 *para*-di-OHs flavonoids (i.e., 5,8-dihydroxy-flavonoids) have been documented: rhodionin [36], 5,8-dihydroxy-3,6,7-trimethoxyflavone [37], 5,8-dihydroxy-6,7-dimethoxyflavone [37], 5,8-dihydroxy-6,7,4′-trimethoxyflavone, and 5,8-dihydroxy-6,7,3′,4′,5′-pentamethoxyflavone [38]. Thereby, our findings may be of great scientific value. (2) ET *plus* H^+^-transfer reactions can cover several possible mechanisms, such as hydrogen atom transfer (HAT), sequential proton-loss electron-transfer (SPLET), electron transfer−proton transfer (ET−PT), and proton coupled electron transfer (PCET) [35,39]. Because these antioxidant mechanisms are essentially involved in ET and H^+^-transfer reactions. The difference among these mechanisms depends on the sequence and cooperativity [40]. Thus, the net result of all these different antioxidant mechanisms is identical; and the proposed reactions in Figure 4 are basically acceptable. Our proposal is also supported by the recent report concerning phenolic antioxidant reaction with alkylperoxyl radical (ROO•) [41].

### 2.3. Antioxidant Role of Other Substituents: Evidence from the PTIO•-Trapping Assay

As discussed above, other substituents may also affect the antioxidant activity of xanthones. These substituents include isoprenyl, cyclized-isoprenyl, methyl, and glycoside. The isoprenyl substituent frequently appears in xanthones, and seldom occurs in other phenolics (such as flavonoid, lignanoid, coumarin, and stilbene [13,14,23,25,42,43,44,45,46]). As a result, its role has not been analyzed by means of the structure-activity relationship [40]. In the study, γ-mangostin (**2**), an isoprenylated xanthone was found to be superior to its parent compound norathyriol (**8**) at pH 4.5. 

This suggested that the isoprenyl substituent enhances the ET potential. Possibly through hydrolysis, isoprenyl substituent can also be transferred into 3-hydroxy-3-methylbutyl substituent. Thus, 3-hydroxy-3-methylbutyl substituent can also be found in xanthones (e.g., **1**, Figure 3). As shown in Table 1, there was no great difference between garcinone C (**1**) and γ-mangostin (**2**); Thus, the effect of the 3-hydroxy-3-methylbutyl substituent was basically equivalent to that of the isoprenyl substituent. In addition, the cyclized-isoprenyl substituent was also found in xanthone (e.g., **10**, Figure 3). Comparison of the IC_50_ values of 1,3,5,6-tetrahydroxyxanthone (**9**) and isojacareubin (**10**) revealed that the cyclized-isoprenyl substituent decreases the antioxidant activity (Table 1). This may be attributed to the fact that the cyclized-isoprenyl substituent replaces a phenolic -OH at the 3-position.

Like the cyclized-isoprenyl substituent, the methyl substituent may also replace phenolic -OHs. A typical example was mangiferin (**4**) and its ether 7-*O*-methylmangiferin (**14**). As shown in Table 1, the IC_50_ values at two pH values (pH 4.5 and 7.4) decrease significantly from mangiferin (**4**) to 7-*O*-methylmangiferin (**14**). This is because methylation breaks the 6,7-di-OHs construction in mangiferin (**4**).

Like the methyl substituent, the glycoside substituent frequently occurs in phenolic antioxidants (including xanthones). As seen in Table 1, both mangiferin (**4**) and isomangiferin (**12**) can be regarded as the glycosidated derivatives of norathyriol (**8**); and mangiferin (**4**) and isomangiferin (**12**) are actually two positional-isomers of each other. However, in the study, the three xanthones were classified into as the strong antioxidants (Table 1). The fact implies that, the effect of glycoside substituent at any C-position is very limited.

In brief, during the PTIO•-trapping process, the above substituents played a minor role in antioxidant activity. However, when these substituents break the aforementioned four types (*para*-di-OHs, 5,6-di-OHs, 6,7-di-OHs, and 7,8-di-OHs), they can greatly lower the antioxidant activity of xanthones.

## 3. Materials and Methods

### 3.1. Chemicals and Animals

2-Phenyl-4,4,5,5-tetramethylimidazoline-1-oxyl 3-oxide radical (PTIO•, CAS 18390-00-6, >98.0%, M.W. 233.29) was purchased from TCI Chemical Co. (Shanghai, China). (±)-6-Hydroxyl-2,5,7,8-tetramethlychromane-2-carboxylic acid (Trolox, CAS 53188-07-1, 97%, M.W. 250.29) was purchased from Sigma-Aldrich Shanghai Trading Co. (Shanghai, China). Garcinone C (CAS 76996-27-5, C_23_H_26_O_7_, M.W. 414.5, 98%, Supplementary Materials S2) and γ-mangostin (CAS 31271-07-5, C_23_H_24_O_6_, M.W. 396.4, 97%, Supplementary Materials S3) were purchased from Chengdu Biopurify Phytochemicals Ltd. (Chengdu, China). Subelliptenone G (CAS 162473-22-5, C_13_H_8_O_5_, M.W. 244.2, purity 97%, Supplementary Materials S4) was purchased from BioBioPha Co., Ltd. (Kunming, China). Mangiferin (CAS 4773-96-0, C_19_H_18_O_11_, M.W. 422.3, 98%, Supplementary Materials S5) was purchased from Chengdu Biopurify Phytochemicals Ltd. 1,6,7-Trihydroxyxanthone (CAS 25577-04-2, C_13_H_8_O_5_, M.W. 244.2, purity 98%, Supplementary Materials S6), 1,2,5-trihydroxyxanthone (CAS 156640-23-2, C_13_H_8_O_5_, M.W. 244.2, purity 98%, Supplementary Materials S7), 1,5,6-trihydroxyxanthone (CAS 5042-03-5, C_13_H_8_O_5_, M.W. 244.2, purity 98%, Supplementary Materials S8), norathyriol (CAS 3542-72-1, C_13_H_8_O_6_, M.W. 260.2, purity 98%, Supplementary Materials S9), 1,3,5,6-tetrahydroxyxanthone (CAS 5084-31-1, C_13_H_8_O_6_, M.W. 260.2, purity 98%, Supplementary Materials S10) and isojacareubin (CAS 50597-93-8, C_18_H_14_O_6_, M.W. 326.3, purity 97%, Supplementary Materials S11) were purchased from BioBioPha Co., Ltd. 1,3,5,8-Tetrahydroxyxanthone (CAS 2980-32-7, C_13_H_8_O_6_, M.W. 260.2, 98%, Supplementary Materials S12), and isomangiferin (CAS 24699-16-9, C_19_H_18_O_11_, M.W. 422.3, 98%, Supplementary Materials S13) were purchased from Chengdu Biopurify Phytochemicals Ltd. 2-Hydroxyxanthone (CAS 1915-98-6, C_13_H_8_O_3_, M.W. 212.2, purity 98%, Supplementary Materials S14) was purchased from BioBioPha Co., Ltd. 7-*O*-Methylmangiferin (CAS 31002-12-7, C_20_H_20_O_11_, M.W. 436.1, purity 97%, Supplementary Materials S15), neomangiferin (CAS 64809-67-2, C_25_H_28_O_16_, M.W. 584.5, purity 97%, Supplementary Materials S16) and lancerin (CAS 81991-99-3, C_19_H_18_O_10_, M.W. 406.3, purity 98%, Supplementary Materials S17) were purchased from Chengdu Biopurify Phytochemicals Ltd. Other reagents were of analytical grade.

### 3.2. PTIO•-Trapping Colorimetric Assay

The PTIO•-trapping assay was conducted based on our previously published method [10]. The experimental procedures are briefly described as following: PTIO• radical was dissolved in phosphate pH 4.5 and pH 7.4 buffer to prepare PTIO• solution; xanthone samples were prepared using methanol. Various volumes of xanthone methanolic were brought to phosphate pH 4.5 and pH 7.4 buffer, then were mixed with PTIO• solution. After incubation for 12 h, the product mixture ware measured at 560 nm on a microplate reader (Multiskan FC, Thermo Scientific, Shanghai, China). The PTIO• inhibition percentage was calculated as follows:

(1) $$Inhibition\;\%=\frac{A_{0}-A}{A_{0}}\times 100\%$$

where *A*_0_ is the absorbance at 560 nm of the control without the sample, and *A* is the absorbance at 560 nm of the reaction mixture with the sample. The above experiment was repeated using phosphate buffers with different pH values (including pH 4.5 and 7.4).

### 3.3. UPLC−ESI−Q−TOF−MS/MS Analysis of Xanthone Reaction Product with PTIO•

UPLC−ESI−Q−TOF−MS/MS spectra of the reaction products of PTIO• with the xanthone were obtained according to our previously described method [47]. The methanolic solutions of phenolic components were mixed with a solution of PTIO• radicals in methanol at a molar ratio of 1:2, and the resulting mixtures were incubated for 24 h at room temperature. The product mixtures were filtered through a 0.22-μm filter and measured using a UPLC-ESI-Q-TOF-MS/MS system equipped with a C_18_ column (2.0 mm i.d. × 100 mm, 2.2 μm, Shimadzu Co., Kyoto, Japan). The mobile phase was used for elution and consisted of a mixture of methanol (phase A) and water (phase B). The column was eluted at a flow rate of 0.3 mL/min with the following gradient elution program: 0–10 min, 60–100% A; 10–15 min, 100% A. The sample injection volume was set at 1 μL to separate the components, and the column temperature was 40 °C. The Q-TOF-MS/MS analysis was conducted on a Triple TOF 5600+ mass spectrometer (AB SCIEX, Framingham, MA, USA) equipped with an ESI source, which was run in the negative ionization mode. The scan range was set at 50–1600 Da. The system was run with the following parameters: ion spray voltage, −4500 V; ion source heater, 550 °C; curtain gas (CUR, N_2_), 30 psi; nebulizing gas (GS1, air), 50 psi; and TurboIonSpray (TIS) gas (GS2, air), 50 psi. The declustering potential (DP) was set at −100 V, and the collision energy (CE) was set at −40 V with a collision energy spread (CES) of 20 V. The RAF products were quantified by the extracting the corresponding formula (e.g., [C_26_H_14_O_12_-H]^−^ for the norathyriol-norathyriol dimer) from the total ion chromatogram and integrating the corresponding peaks [48,49].

### 3.4. Statistical Analysis

Each experiment was performed in triplicate and the data were recorded as mean ± SD (standard deviation). The dose–response curves were plotted using Origin 6.0 professional software (OriginLab, Northampton, MA, USA). The IC_50_ value was defined as the final concentration of 50% radical inhibition (or relative reducing power) [50]. It was calculated by linear regression analysis, and expressed as the mean ± SD (*n* = 3). The linear regression was analyzed using Origin 6.0. Determination of significant differences between the mean IC_50_ values was performed using one-way ANOVA and the *t*-test. The analysis was performed using SPSS software 13.0 for Windows (SPSS Inc., Chicago, IL, USA). *p* < 0.05 was considered to be statistically significant.

## 4. Conclusions

Phenolic xanthones may react via ET *plus* H^+^-transfer to present antioxidant activity. Through these mechanisms, most xanthones can further dimerize unless they bear huge substituents with steric hindrance. The antioxidant activity of phenolic xanthones is governed by any of four substituent types, i.e., *para*-di-OHs, 5,6-di-OHs, 6,7-di-OHs, and 7,8-di-OHs. The effects of other types of substituents are very limited. In general, isoprenyl and 3-hydroxy-3-methylbutyl substituents can slightly enhance ET potential. Cyclized-isoprenyl, methyl, and glycoside substituents can cut down phenolic -OH to lower antioxidant activity. However, if these substituents break the aforementioned four types, their detrimental effect may be enhanced.

## Figures and Tables

**Table 1:** The main results of PTIO•-trapping activity of 16 xanthones using UPLC-ESI-Q-TOF-MS/MS analysis and colorimetric analysis.

No	Xanthone	Main Results of UPLC-ESI-Q-TOF-MS/MS Analysis of Reaction Products				IC_50_ in Colorimetric Analysis/μM		Activity
		Retention Time/Min	Primary MS Spectra	MS/MS Spectra	Product	pH 4.5	pH 7.4	
**1**	Garcinone C	5.961	825, 826, 827	353, 411, 825	dimer	36.0 ± 3.1	40.8 ± 2.0	Strong
**2**	γ-Mangostin	9.847	789, 790, 791	375, 393, 394, 395, 677	dimer	45.5 ± 2.4	60.4 ± 1.8	
**3**	Subelliptenone G	1.739	487, 488	No data	dimer	63.4 ± 13.3	110.9 ± 3.8	
**4**	Mangiferin	1.105	841, 842, 843, 844	329, 419, 601, 631, 661, 721,751, 823	dimer	64.1 ± 8.5	38.0 ± 2.7	
**5**	1,6,7-Trihydroxyxanthone	1.813	485, 846, 487	243, 349	dimer	83.0 ± 2.2	90.3 ± 3.0	
**6**	1,2,5-Trihydroxyxanthone	1.648	487, 488	No data	dimer	89.1 ± 7.4	112.2 ± 0.2	
**7**	1,5,6-Trihydroxyxanthone	2.044	485, 486, 487, 488	243, 485	dimer	101.3 ± 16.6	116.3 ± 2.2	
**8**	Norathyriol	1.341	517, 518, 519, 520	229, 257, 258, 259, 365, 499, 517	dimer	103.0 ± 3.7	54.1 ± 0.9	
**9**	1,3,5,6-Tetrahydroxyxanthone	1.349	517, 518, 519, 520	229, 257, 258, 259, 365, 499, 517	dimer	108.1 ± 19.4	102.7 ± 4.7	
**10**	Isojacareubin	8.483	649, 650, 651, 652	323-325, 649	dimer	108.7 ± 0.1	136.7 ± 7.3	
**11**	1,3,5,8-Tetrahydroxyxanthone	1.726	519, 520, 521	215, 259, 260	dimer	116.7 ± 12.6	133.1 ± 29.4	
**12**	Isomangiferin	1.105	841, 842, 843, 844	329, 419, 601, 631, 661, 721,751, 823	dimer	121.3 ± 8.3	104.1 ± 12.3	
**13**	2-Hydroxyxanthone	1.885	423, 424	No data	dimer	284.2 ± 48.8	142.5 ± 13.1	Weak
**14**	7-*O*-Methylmangiferin	1.204	871, 872	No data	dimer	387.2 ± 37.5	234.9 ± 1.7	
**15**	Neomangiferin	No product	No product	No product	No product	545.2 ± 15.2	213.1 ± 23.8	
**16**	Lancerin	1.165–1.191	811, 812	No date	dimer	681.2 ± 7.9	1106.3 ± 202.6	

The IC_50_ value was defined as the final concentration of 50% PTIO• radical inhibition and was calculated by linear regression analysis and expressed as the mean ± SD (*n* = 3). The linear regression was analyzed using Origin 6.0 professional software. Trolox is the positive control. Its IC_50_ values were calculated as 187.5 ± 24.2 μM (pH 4.5) and 175.0 ± 12.4 μM (pH 7.4). The dose-response curves are listed in Supplementary Materials S1; while the original UPLC-ESI-Q-TOF-MS/MS data were listed in Supplementary Materials S18. The 16 xanthones were classified based on the comparison of IC_50_ values at pH 4.5. These data were analyzed by independent *t*-test for comparison between two groups. Multiple comparisons within the same group was conducted by one-way ANOVA. *p* < 0.05 was considered statistically significant. The statistical analysis was performed using SPSS 11.5 system (SPSS, Chicago, IL, USA).

## Supplementary Materials

The following are available online. Supplementary Materials S1 Dose response curves and IC_50_ values; Supplementary Materials S2–17 Analysis certificates and appearances of 16 xanthones. Supplementary Materials S18 Original MS spectra; Supplementary Materials S19 dimerization reaction of norathyriol and MS spectra elucidation; Supplementary Materials S20 dimerization reaction of 1,3,5,6-tetrahydroxyxanthone and MS spectra elucidation.

## Abbreviations

The following abbreviations are used in this manuscript:

	
	
ABTS•^+^	2,2′-azino-bis(3-ethylbenzothiazoline-6-sulphonic acid)
DPPH•	1,1-diphenyl-2-picryl-hydrazl radical
ET	electron transfer
ET-PT	electron transfer−proton transfer
HAT	hydrogen atom transfer
PCET	proton coupled electron transfer
PTIO•	2-phenyl-4,4,5,5-tetramethylimidazoline-1-oxyl 3-oxide radical
RAF	radical adduct formation
ROS	reactive oxygen species
SD	standard deviation
SPLET	sequential proton-loss electron-transfer
Trolox	(±)-6-hydroxy-2,5,7,8-tetramethlychromane-2-carboxylic acid
UPLC−ESI−Q−TOF−MS/MS	ultra-performance liquid chromatography coupled with electrospray ionization quadrupole time-of-flight tandem mass spectrometry
