# Supplementary material for: 2-Phenyl-4,4,5,5-tetramethylimidazoline-1-oxyl 3-oxide Radical (PTIO•) Trapping Activity and Mechanisms of 16 Phenolic Xanthones
Source: Molecules. 2018 Jul 11;23(7):1692. doi: 10.3390/molecules23071692 (PMC6100357; doi:10.3390/molecules23071692)
Supplement: Supplementary file 1 [file molecules-23-01692-s001.zip › Suppl/Suppl. 1 Dose response curves IC50 values.docx]

**Supplemental Material-1 Dose response curves and IC_50_ values**

2-Phenyl-4,4,5,5-tetramethylimidazoline-1-oxyl 3-oxide radical (PTIO•) Trapping Activity and Mechanisms of 16 Phenolic Xanthones

Xican Li ^1, 2, †, *^, Ban Chen ^1, 2, †^, Xiaojun Zhao ^1, 2^, Dongfeng Chen ^3, 4, *^

^1^ School of Chinese Herbal Medicine, Guangzhou University of Chinese Medicine, Waihuan East Road No. 232, Guangzhou Higher Education Mega Center, Guangzhou 510006, China; imchenban@foxmail.com (B.C.)

^2^ Innovative Research & Development Laboratory of TCM, Guangzhou University of Chinese Medicine, Waihuan East Road No. 232, Guangzhou Higher Education Mega Center, Guangzhou 510006, China;

^3^ School of Basic Medical Science, Guangzhou University of Chinese Medicine, Waihuan East Road No. 232, Guangzhou Higher Education Mega Center, Guangzhou 510006, China;

^4^ The Research Center of Basic Integrative Medicine, Guangzhou University of Chinese Medicine, Waihuan East Road No. 232, Guangzhou Higher Education Mega Center, Guangzhou 510006, China;

***** Correspondence: lixican@126.com or lixc@gzucm.edu.cn (X.L.); [chen888@gzucm.edu.cn](mailto:chen888@gzucm.edu.cn) (D.C.);
Tel.: +86-203-935-8076 (X.L.)

**^†^** These authors contributed equally to this work.

**Note:** This Supplemental Material provides the Dose response curves and IC50 values. The data with underline are cited in **Table 1** in the main text.

Figure S1**:** The dose response curves of garcinone C in PTIO• scavenging assay at pH 4.5 (A) and 7.4 (B). The value is expressed as mean ± SD (n = 3).

Table. S1 The IC_50_ values of garcinone C in PTIO• scavenging assay, μg/mL (μM).

|  | pH 4.5 | pH 7.4 |
| --- | --- | --- |
| Garcinone C | 14.9±1.3  (36.0±3.1) | 16.9±0.8  (40.8±2.0) |
|  |  |  |

The IC_50_ value (μg/mL) was obtained from Figure S1. The value is expressed as mean ± SD (n = 3).

Figure S2**:** The dose response curves of γ-mangostin in PTIO• scavenging assay at pH 4.5 (A) and 7.4 (B). The value is expressed as mean ± SD (n = 3).

Table. S2 The IC_50_ values of γ-mangostin in PTIO• scavenging assay, μg/mL (μM).

|  | pH 4.5 | pH 7.4 |
| --- | --- | --- |
| γ-Mangostin | 18.0±0.9  (45.5±2.4) | 23.9±0.7  (60.4±1.8) |
|  |  |  |

The IC_50_ value (μg/mL) was obtained from Figure S2. The value is expressed as mean ± SD (n = 3).

Figure S3**:** The dose response curves of subelliptenone G in PTIO• scavenging assay at pH 4.5 (A) and 7.4 (B). The value is expressed as mean ± SD (n = 3).

Table. S3 The IC_50_ values of subelliptenone G in PTIO• scavenging assay, μg/mL (μM).

|  | pH 4.5 | pH 7.4 |
| --- | --- | --- |
| Subelliptenone G | 15.5±3.2  (63.4±13.3) | 27.0±0.9  (110.9±3.8) |
|  |  |  |

The IC_50_ value (μg/mL) was obtained from Figure S3. The value is expressed as mean ± SD (n = 3).

Figure S4**:** The dose response curves of mangiferin in PTIO• scavenging assay at pH 4.5 (A) and 7.4 (B). The value is expressed as mean ± SD (n = 3).

Table. S4 The IC_50_ values of mangiferin in PTIO• scavenging assay, μg/mL (μM).

|  | pH 4.5 | pH 7.4 |
| --- | --- | --- |
| Mangiferin | 27.1±3.6  (64.1±8.5) | 16.0±1.2  (38.0±2.7) |
|  |  |  |

The IC_50_ value (μg/mL) was obtained from Figure S4 The value is expressed as mean ± SD (n = 3).

Figure S5**:** The dose response curves of 1,6,7-trihydroxyxanthone in PTIO• scavenging assay at pH 4.5 (A) and 7.4 (B). The value is expressed as mean ± SD (n = 3).

Table. S5 The IC_50_ values of 1,6,7-trihydroxyxanthone in PTIO• scavenging assay, μg/mL (μM).

|  | pH 4.5 | pH 7.4 |
| --- | --- | --- |
| 1,6,7-Trihydroxyxanthone | 20.2±0.5  (83.0±2.2) | 22.0±0.7  (90.3±3.0) |
|  |  |  |

The IC_50_ value (μg/mL) was obtained from Figure S5. The value is expressed as mean ± SD (n = 3).

Figure S6**:** The dose response curves of 1,2,5-trihydroxyxanthone in PTIO• scavenging assay at pH 4.5 (A) and 7.4 (B). The value is expressed as mean ± SD (n = 3).

Table. S6 The IC_50_ values of 1,2,5-trihydroxyxanthone in PTIO• scavenging assay, μg/mL (μM).

|  | pH 4.5 | pH 7.4 |
| --- | --- | --- |
| 1,2,5-Trihydroxyxanthone | 21.8±1.8  (89.1±7.4) | 27.5±0.1  (112.5±0.2) |
|  |  |  |

The IC_50_ value (μg/mL) was obtained from Figure S6. The value is expressed as mean ± SD (n = 3).

Figure S7**:** The dose response curves of 1,5,6-trihydroxyxanthone in PTIO• scavenging assay at pH 4.5 (A) and 7.4 (B). The value is expressed as mean ± SD (n = 3).

Table. S7 The IC_50_ values of 1,5,6-trihydroxyxanthone in PTIO• scavenging assay, μg/mL (μM).

|  | pH 4.5 | pH 7.4 |
| --- | --- | --- |
| 1,5,6-Trihydroxyxanthone | 24.7±4.1  (101.3±16.6) | 28.4±0.5  (116.3±2.2) |
|  |  |  |

The IC_50_ value (μg/mL) was obtained from Figure S7. The value is expressed as mean ± SD (n = 3).

Figure S8**:** The dose response curves of norathyriol in PTIO• scavenging assay at pH 4.5 (A) and 7.4 (B). The value is expressed as mean ± SD (n = 3).

Table. S8 The IC_50_ values of norathyriol in PTIO• scavenging assay, μg/mL (μM).

|  | pH 4.5 | pH 7.4 |
| --- | --- | --- |
| Norathyriol | 23.5±0.8  (103.0±3.7) | 12.2±0.2  (54.1±0.9) |
|  |  |  |

The IC_50_ value (μg/mL) was obtained from Figure S8. The value is expressed as mean ± SD (n = 3).

Figure S9**:** The dose response curves of 1,3,5,6-tetrahydroxyxanthone in PTIO• scavenging assay at pH 4.5 (A) and 7.4 (B). The value is expressed as mean ± SD (n = 3).

Table. S9 The IC_50_ values of 1,3,5,6-tetrahydroxyxanthone in PTIO• scavenging assay, μg/mL (μM).

|  | pH 4.5 | pH 7.4 |
| --- | --- | --- |
| 1,3,5,6-Tetrahydroxyxanthone | 28.1±5.0  (108.1±19.4) | 26.7±1.2  (102.7±4.7) |
|  |  |  |

The IC_50_ value (μg/mL) was obtained from Figure S9. The value is expressed as mean ± SD (n = 3).

Figure S10**:** The dose response curves of isojacareubin in PTIO• scavenging assay at pH 4.5 (A) and 7.4 (B). The value is expressed as mean ± SD (n = 3).

Table. S10 The IC_50_ values of isojacareubin in PTIO• scavenging assay, μg/mL (μM).

|  | pH 4.5 | pH 7.4 |
| --- | --- | --- |
| Isojacareubin | 35.5±0.1  (108.7±0.1) | 44.6±2.4  (136.7±7.3) |
|  |  |  |

The IC_50_ value (μg/mL) was obtained from Figure S10. The value is expressed as mean ± SD (n = 3).

Figure S11**:** The dose response curves of 1,3,5,8-tetrahydroxyxanthone in PTIO• scavenging assay at pH 4.5 (A) and 7.4 (B). The value is expressed as mean ± SD (n = 3).

Table. S11 The IC_50_ values of 1,3,5,8-tetrahydroxyxanthone in PTIO• scavenging assay, μg/mL (μM).

|  | pH 4.5 | pH 7.4 |
| --- | --- | --- |
| 1,3,5,8-Tetrahydroxyxanthone | 30.3±3.3  (116.7±12.6) | 34.6±7.6  (133.1±29.4) |
|  |  |  |

The IC_50_ value (μg/mL) was obtained from Figure S11. The value is expressed as mean ± SD (n = 3).

Figure S12**:** The dose response curves of isomangiferin in PTIO• scavenging assay at pH 4.5 (A) and 7.4 (B). The value is expressed as mean ± SD (n = 3).

Table. S12 The IC_50_ values of isomangiferin in PTIO• scavenging assay, μg/mL (μM).

|  | pH 4.5 | pH 7.4 |
| --- | --- | --- |
| Isomangiferin | 51.2±3.5  (121.3±8.3) | 44.0±5.2  (104.1±12.3) |
|  |  |  |

The IC_50_ value (μg/mL) was obtained from Figure S12. The value is expressed as mean ± SD (n = 3).

Figure S13**:** The dose response curves of 2-hydroxyxanthone in PTIO• scavenging assay at pH 4.5 (A) and 7.4 (B). The value is expressed as mean ± SD (n = 3).

Table. S13 The IC_50_ values of 2-hydroxyxanthone in PTIO• scavenging assay, μg/mL (μM).

|  | pH 4.5 | pH 7.4 |
| --- | --- | --- |
| 2-Hydroxyxanthone | 60.3±10.3  (284.2±48.8) | 30.2±2.8  (142.5±13.1) |
|  |  |  |

The IC_50_ value (μg/mL) was obtained from Figure S13. The value is expressed as mean ± SD (n = 3).

Figure S14**:** The dose response curves of 7-O-methylmangiferin in PTIO• scavenging assay at pH 4.5 (A), 6.0 (B) and 7.4 (C). The value is expressed as mean ± SD (n = 3).

Table. S14 The IC_50_ values of 7-O-methylmangiferin in PTIO• scavenging assay, μg/mL (μM).

|  | pH 4.5 | pH 7.4 |
| --- | --- | --- |
| 7-O-Methylmangiferin | 168.8±16.3  (387.2±37.5) | 102.4±0.8  (234.9±1.7) |
|  |  |  |

The IC_50_ value (μg/mL) was obtained from Figure S14. The value is expressed as mean ± SD (n = 3).

Figure S15**:** The dose response curves of neomangiferin in PTIO• scavenging assay at pH 4.5 (A) and 7.4 (B). The value is expressed as mean ± SD (n = 3).

Table. S15 The IC_50_ values of neomangiferin in PTIO• scavenging assay, μg/mL (μM).

|  | pH 4.5 | pH 7.4 |
| --- | --- | --- |
| Neomangiferin | 318.4±8.9  (545.2±15.2) | 124.5±13.9  (213.1±23.8) |
|  |  |  |

The IC_50_ value (μg/mL) was obtained from Figure S15. The value is expressed as mean ± SD (n = 3).

Figure S16**:** The dose response curves of lancerin in PTIO• scavenging assay at pH 4.5 (A) and 7.4 (B). The value is expressed as mean ± SD (n = 3).

Table. S16 The IC_50_ values of lancerin in PTIO• scavenging assay, μg/mL (μM).

|  | pH 4.5 | pH 7.4 |
| --- | --- | --- |
| Lancerin | 276.6±3.2  (681.2±7.9) | 449.2±82.2  (1106.3±202.6) |
|  |  |  |

The IC_50_ value (μg/mL) was obtained from Figure S16. The value is expressed as mean ± SD (n = 3).

Figure S17**:** The dose response curves of Trolox in PTIO• scavenging assay at pH 4.5 (A) and 7.4 (B). The value is expressed as mean ± SD (n = 3).

Table. S17 The IC_50_ values of Trolox in PTIO• scavenging assay, μg/mL (μM).

|  | pH 4.5 | pH 7.4 |
| --- | --- | --- |
| Trolox | 46.9±6.1  (187.5±24.2) | 43.8±3.1  (175.0±12.4) |
|  |  |  |

The IC_50_ value (μg/mL) was obtained from Figure S17. The value is expressed as mean ± SD (n = 3).
