# Supplementary material for: 2-Phenyl-4,4,5,5-tetramethylimidazoline-1-oxyl 3-oxide Radical (PTIO•) Trapping Activity and Mechanisms of 16 Phenolic Xanthones
Source: Molecules. 2018 Jul 11;23(7):1692. doi: 10.3390/molecules23071692 (PMC6100357; doi:10.3390/molecules23071692)
Supplement: Supplementary file 1 [file molecules-23-01692-s001.zip › Suppl/Suppl. 11 Appearance and analysis certificate of isojacareubin.pdf]

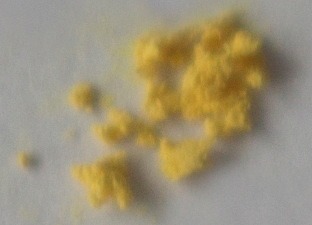

Isojacarebuin

西力生物
BBP BioBioPha

Natural Product Library for Drug Discovery

CERTIFICATE OF ANALYSIS

BBP No.: BBP03640

CAS No.: 50597-93-8

Chemical Name: Isojacareubin

Molecular Formula: $C_{18}H_{14}O_6$

Structure:

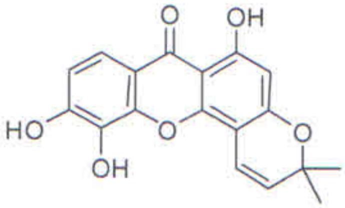

Purity: 97%

Appearance: Orange powder

Solvent: Dimethyl sulfoxide, methanol

Exact Weight: 5.1 mg

Storage: Store in a dark place under the temperature of 0−4 °C

Intended Use: For laboratory use only

Reference: Q. L. Wu, Phytochemistry, 1998, 49(5), 1395-1402

Warm Notice: When publishing, please cite as: ***chemical name*** was purchased from BioBioPha Co., Ltd. (Kunming, China)

Characterization Data Summary

Analytical Test	Results
Identification by ^{1}H-NMR	Consistent with the above structure
Purity tested by HPLC, ^{1}H-NMR	97%

Authorized Signature: BioBioPha Co., Ltd. 2018.04.20 PASS

Date:

Tel.: +86 871 6521 7109 Fax: +86 871 6521 5563 E-mail: sales@mail.biobiopha.com

西力生物
BBP BioBioPha

Natural Product Library for Drug Discovery

PRODUCT QUALITY REPORT

Product Number: BBP03640

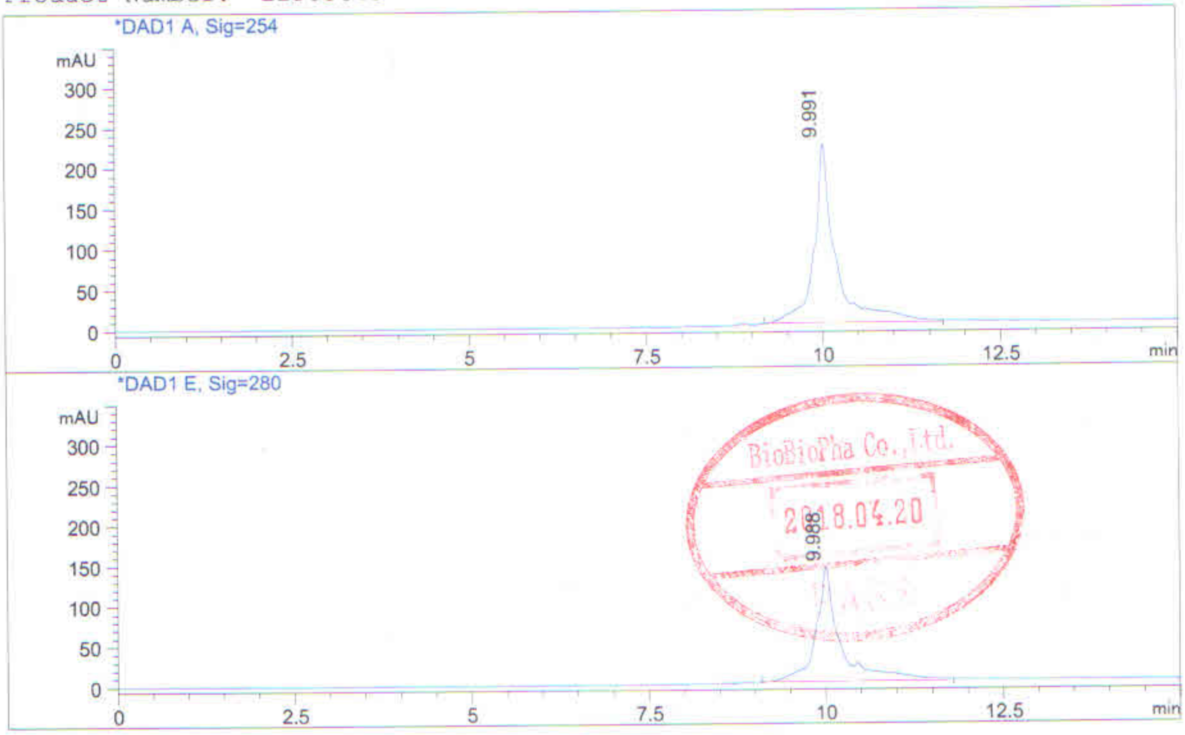

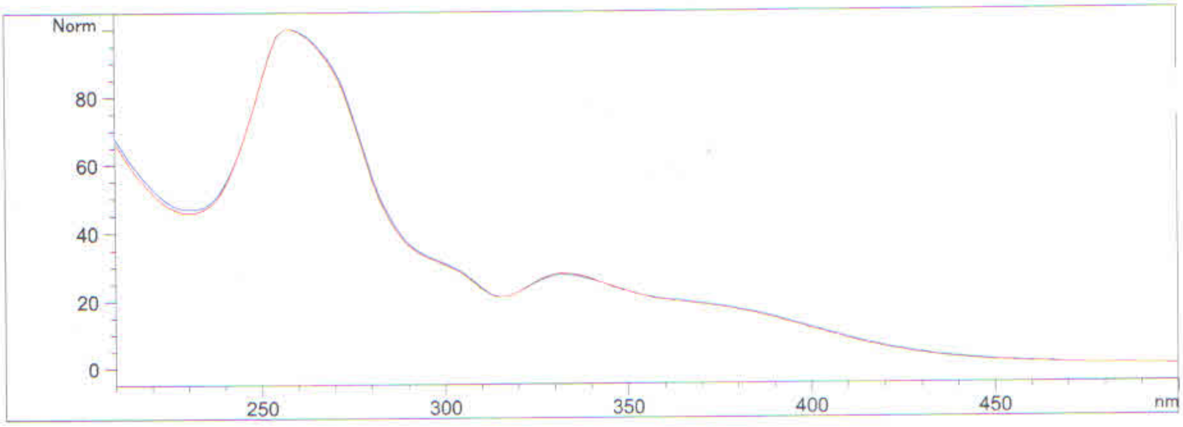

Agilent 1200 series HPLC system
InertSustain C18 column (5 μm, 4.6 × 150 mm)
20%→100% MeOH in H_2O over 8.0 min followed by 100% MeOH to 15.0 min
1.0 ml/min, 20°C

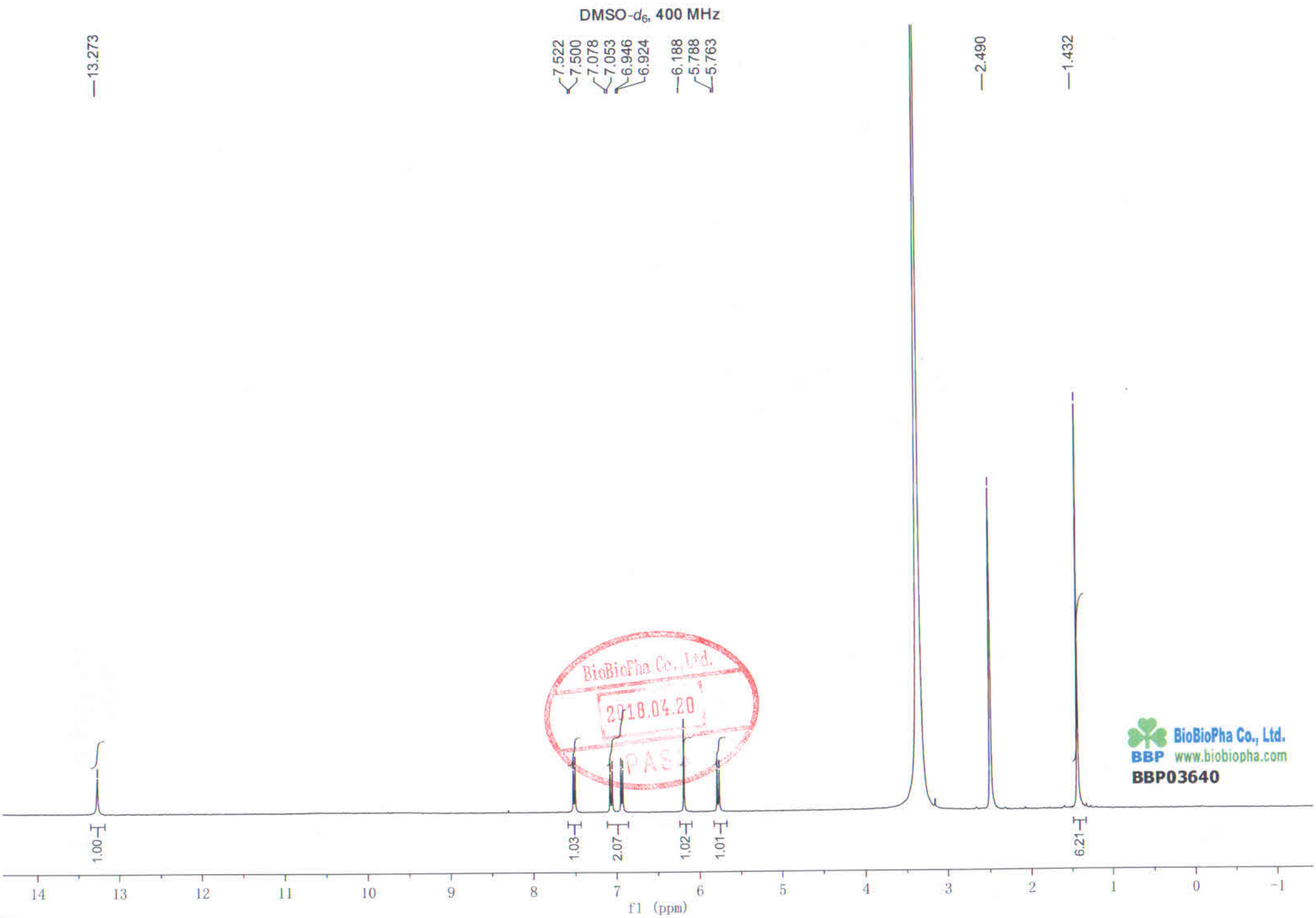
DMSO-d_6, 400 MHz
13.273
7.522
7.500
7.078
7.053
6.946
6.924
6.188
5.788
5.763
2.490
1.432
1.00
1.03
2.07
1.02
1.01
6.21
14 13 12 11 10 9 8 7 6 5 4 3 2 1 0 -1
f1 (ppm)
BioBioPha Co., Ltd.
2018.04.20
PASS
BioBioPha Co., Ltd.
BBP www.biobiopha.com
BBP03640
