# Supplementary material for: 2-Phenyl-4,4,5,5-tetramethylimidazoline-1-oxyl 3-oxide Radical (PTIO•) Trapping Activity and Mechanisms of 16 Phenolic Xanthones
Source: Molecules. 2018 Jul 11;23(7):1692. doi: 10.3390/molecules23071692 (PMC6100357; doi:10.3390/molecules23071692)
Supplement: Supplementary file 1 [file molecules-23-01692-s001.zip › Suppl/Suppl. 12 Appearance and analysis certificate of 1,3,5,8-tetrahydroxyxanthone.pdf]

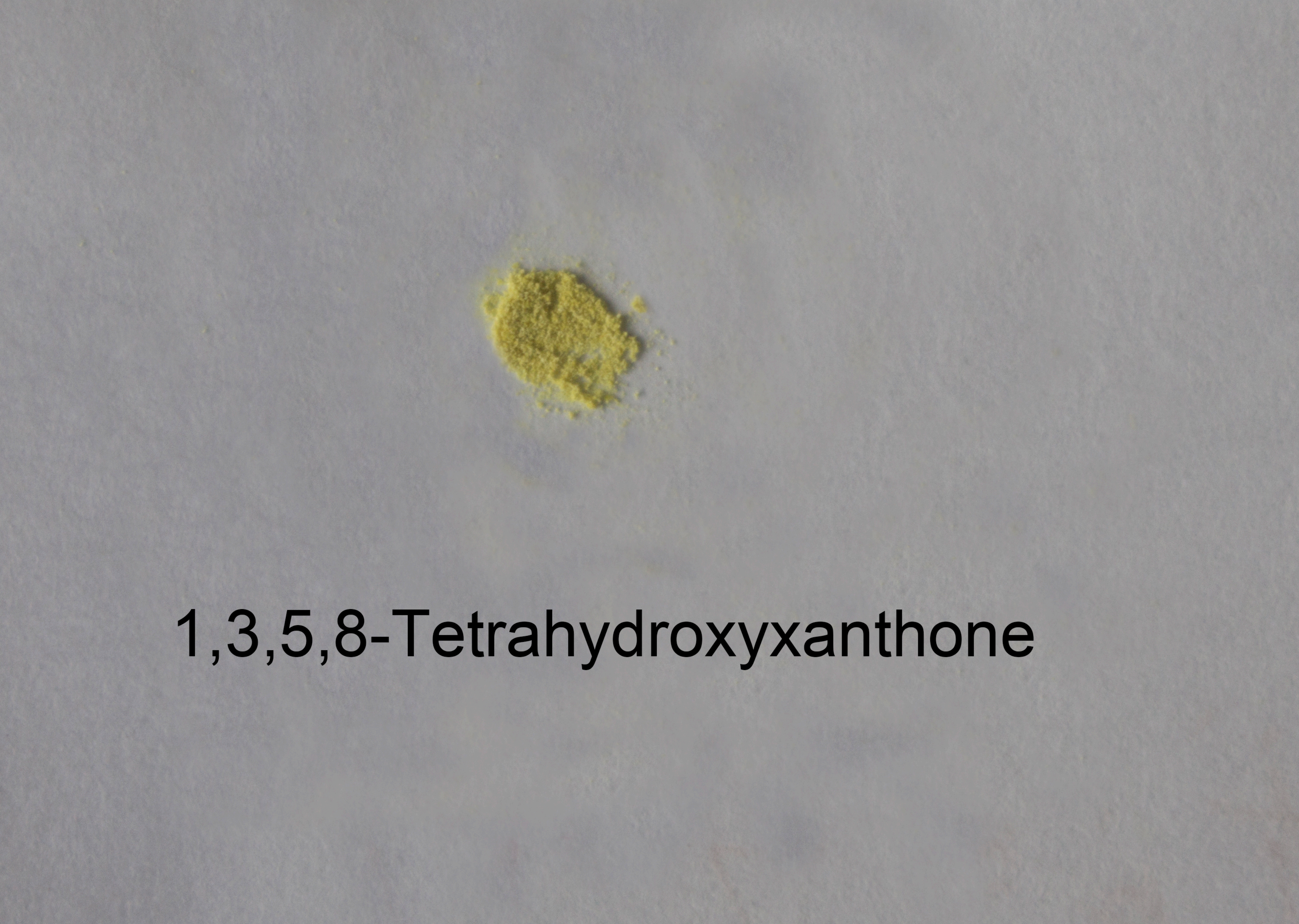
1,3,5,8-Tetrahydroxyxanthone

成都普瑞法科技开发有限公司
Chengdu Biopurify Phytochemicals Ltd.

Add: No.11 Building, No. 388 Rongtaidadao CNSTP
Wenjiang Zone, Chengdu, Sichuan, 611130 China
TEL: 028-82633987 FAX: 028-82633165
E-mail: biopurify@gmail.com sales@biopurify.com
Web: www.biopurify.com

Certificate of Analysis

Product Name: 1,3,5,8-Tetrahydroxyxanthone

Other Name:

Structure:

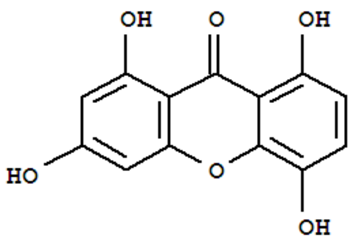

Catalogue No.: BP0003

Batch No.: 15121702

Reported Date: 2015-12-17

CAS Number: 2980-32-7

Mol. Formula: $C_{13}H_8O_6$

Mol. Weight: 260.201

Botanical Source:

Type of compound:

Identification Method: Mass, NMR

Analysis Method of Purity: HPLC-DAD

www.biopurify.cc

Analytical result:

Test	Specification	Results
Appearance	Pale Yellow powder	Pale Yellow powder
Loss on drying	＜3.0%	1.3%
Purity (HPLC, 254nm)*	≥92.0%	99.12%

***** Please find HPLC chromatography attached.

Package: Brown vial or HDPE Plastic Bottle

Storage: Cool and Dry place, protected from light, keep package airproofed when not in use.

Expiration: two years (2017-12-17) under conditions list above.

QC: Meng Pan
Date: 2015-12-17

QA: Lianglei Zhang
Date: 2015-12-17

成都普瑞法科技开发有限公司
Chengdu Biopurify Phytochemicals Ltd.

Empower2 software

Chengdu Biopurify Phytochemicals Ltd

SAMPLE INFORMATION

Sample Name:	Demethylbellidifolin15121702	Acquired By:	panmeng
Sample Type:	Unknown	Sample Set Name:	
Vial:	1:F,1	Acq. Method Set:	Demethylbellidifolin
Injection #:	1	Processing Method:	Samples
Injection Volume:	10.00 ul	Channel Name:	254.0nm
Run Time:	20.0 Minutes	Proc. Chnl. Descr.:	PDA 254.0 nm

Date Acquired: 2015-12-17 11:31:57 CST
Date Processed: 2015-12-17 12:31:01 CST

Auto-Scaled Chromatogram

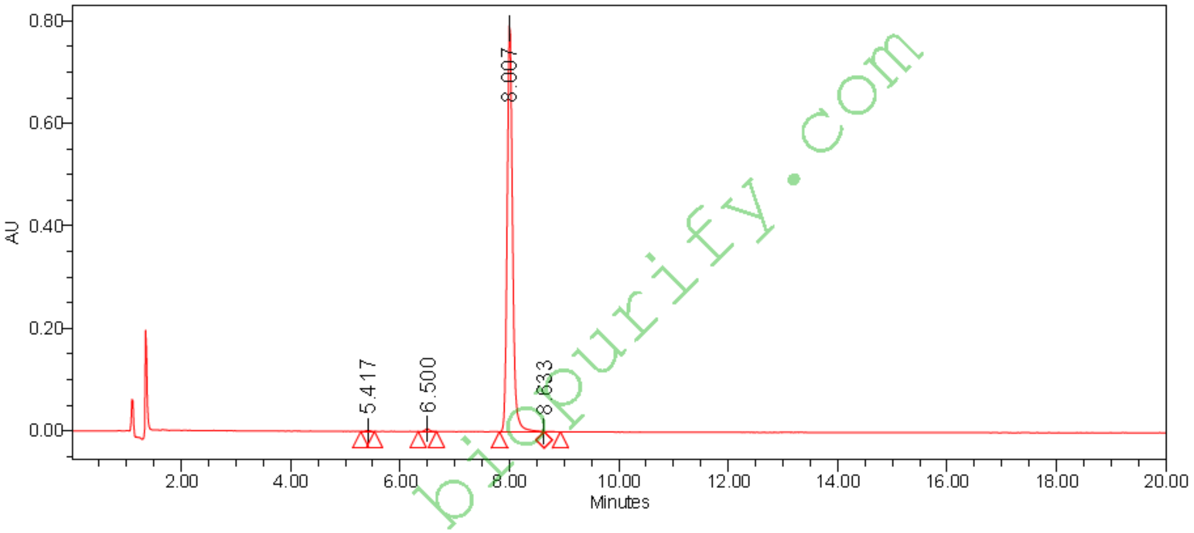

www.biopurify.com

Peak Results

	Name	RT	Area	% Area	USP Plate Count	USP Resolution
1		5.417	11228	0.20	19251.59	
2		6.500	30530	0.55	22818.36	6.54
3		8.007	5474846	99.12	32801.92	8.60
4		8.633	6787	0.12		3.53
