# Supplementary material for: 2-Phenyl-4,4,5,5-tetramethylimidazoline-1-oxyl 3-oxide Radical (PTIO•) Trapping Activity and Mechanisms of 16 Phenolic Xanthones
Source: Molecules. 2018 Jul 11;23(7):1692. doi: 10.3390/molecules23071692 (PMC6100357; doi:10.3390/molecules23071692)
Supplement: Supplementary file 1 [file molecules-23-01692-s001.zip › Suppl/Suppl. 13 Appearance and analysis certificate of isomangiferin.pdf]

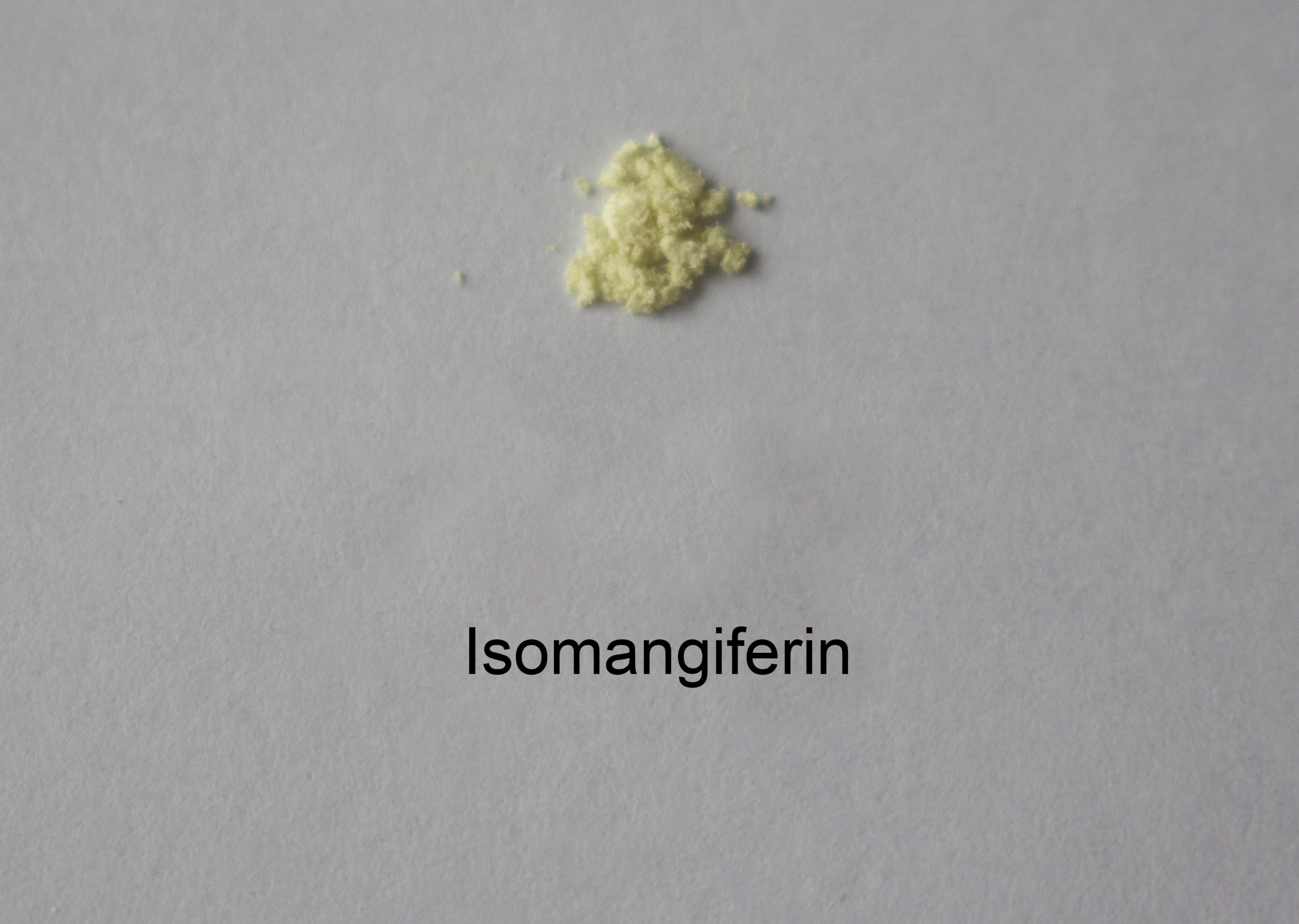
Isomangiferin

产品分析证书
Certificate of Analysis

中文名称：异芒果苷

English Name：Isomangiferin
别名(Alias): 4-Glucosyl-1,3,6,7-tetrahydroxyxanthone

CAS Number: 24699-16-9
分子式(M. F.): $C_{19}H_{18}O_{11}$
分子量(M. W.): 422.342

批号(Batch No.): PRF7082943
报告日期(Report date): 2016/8/29

Structure

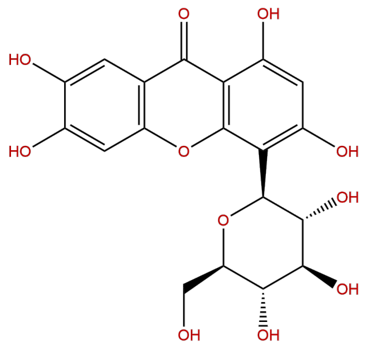

检验结果（Analytical result）：

**检验项目（Test Item）**	**检验指标（Specifications）**	**检验结果（Results）**
外观Appearance	Light yellow powder	Light yellow powder
干燥失重Loss on drying	＜3.0%	1.3 %
纯度Purity (HPLC-DAD, 316nm)*	≥98.0%	99.77%
质谱Mass	422.342±1	Conforms
核磁NMR	Comply with the structure	Conforms

*** 色谱图见附件（Please find HPLC chromatography attached.）**

贮存条件（Storage）:2~8℃
复测期（Retest date）:two years (2018-08-28) under conditions list above.
备注（Remarks）：如遇质量问题，请于收到产品之日起 15 日内与我们联系。
In case of quality issue, please contact us within 15 days after receipt of the product.

QC: Zhang Ling　　　　QA: Wu Qi

Date: 2016年8月29日　　　　Date: 2016年8月29日

SAMPLE INFORMATION

Sample Name:	Isomangiferin PRF7082943	Acquired By:	System
Sample Type:	Unknown	Sample Set Name:	
Vial:	70	Acq. Method Set:	Isomangiferin
Injection #:	1	Processing Method:	sample
Injection Volume:	5.00 ul	Channel Name:	316.0nm
Run Time:	22.0 Minutes	Proc. Chnl. Descr.:	PDA 316.0 nm
Date Acquired:	2016-8-29 15:55:35 CST		
Date Processed:	2016-8-29 16:19:57 CST		

Auto-Scaled Chromatogram

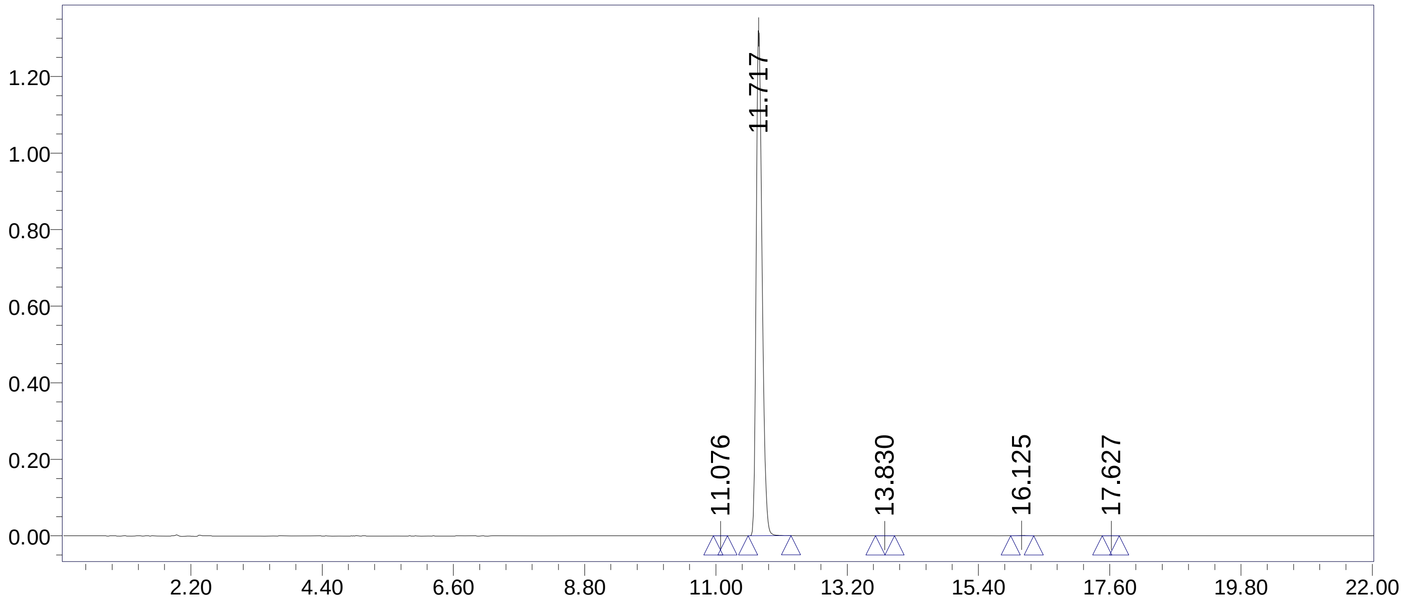

Peak Results

	RT	Area	% Area	USP Plate Count	USP Resolution
1	11.076	2130	0.02	76463.85	
2	11.717	9192060	99.77	63981.30	3.60
3	13.830	3650	0.04	69207.66	10.39
4	16.125	10732	0.12	131642.98	11.50
5	17.627	4606	0.05	188484.35	8.64
