# Supplementary material for: 2-Phenyl-4,4,5,5-tetramethylimidazoline-1-oxyl 3-oxide Radical (PTIO•) Trapping Activity and Mechanisms of 16 Phenolic Xanthones
Source: Molecules. 2018 Jul 11;23(7):1692. doi: 10.3390/molecules23071692 (PMC6100357; doi:10.3390/molecules23071692)
Supplement: Supplementary file 1 [file molecules-23-01692-s001.zip › Suppl/Suppl. 15 Appearance and analysis certificate of 7-O-methylmangiferin.pdf]

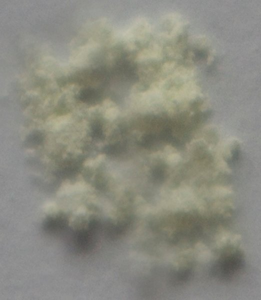

7-O-Methylmangiferin

成都普瑞法科技开发有限公司
Chengdu Biopurify Phytochemicals Ltd.

Add: No.11 Building, No. 388 Rongtaidadao CNSTP
Wenjiang Zone, Chengdu, Sichuan, 611130 China
TEL: 028-82633987 FAX: 028-82633165
E-mail: biopurify@gmail.com sales@biopurify.com
Web: www.biopurify.com

Certificate of Analysis

Product Name: 7-O-Methylmangiferin

Other Name:

Catalogue No.: BP1566

Batch No.: PRF7060808

Reported Date: 2016-06-08

CAS Number: 31002-12-7

Mol. Formula: $C_{20}H_{20}O_{11}$

Mol. Weight: 436.369

Analysis Method of Purity: HPLC-DAD

Structure:

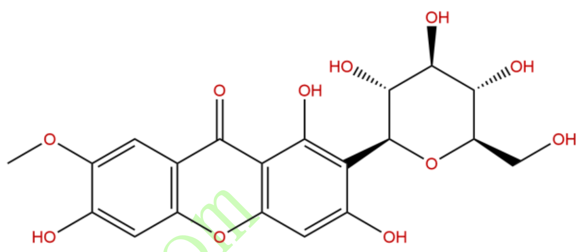

Analytical result:

**Test**	**Specification**	**Results**
Appearance	Pale yellow powder	Pale yellow powder
Loss on drying	＜3.0%	1.3%
Purity (HPLC, 320nm)*	≥98.0%	99.56%

* Please find HPLC chromatography attached.

Package: Brown vial or HDPE Plastic Bottle

Storage: Cool and Dry place, protected from light, keep package airproofed when not in use.

Expiration: two years (2018-06-08) under conditions list above**.**

QC: MengPan **QA:** Lianglei Zhang

Date: 2016-06-08 **Date: 2016-06-08**

www.biopurify.com

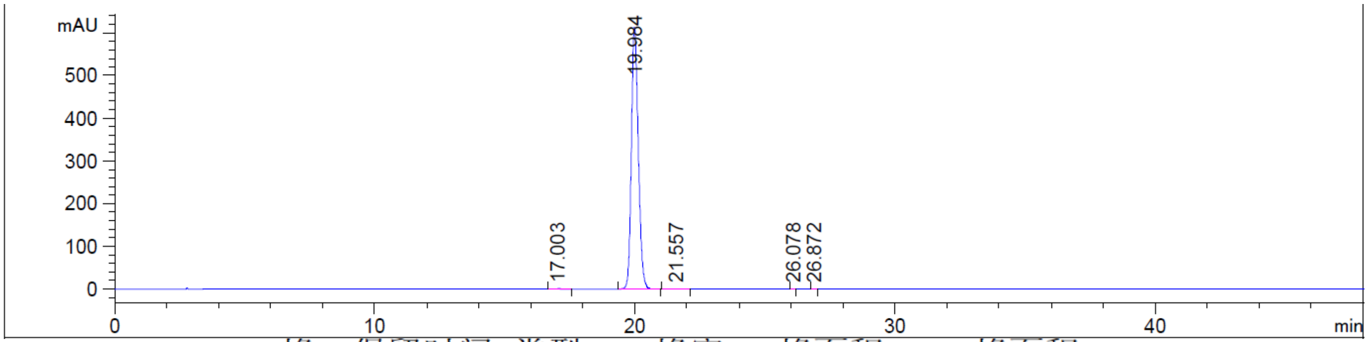

峰 #	保留时间 [min]	类型	峰宽 [min]	峰面积 [mAU*s]	峰面积 %
1	17.003	BB	0.2705	10.30797	0.0902
2	19.984	BB	0.2887	1.13778e4	99.5688
3	21.557	BB	0.2849	30.55885	0.2674
4	26.078	BB	0.0736	5.57325	0.0488
5	26.872	BB	0.0730	2.83672	0.0248

www.biopurify.
