# Supplementary material for: 2-Phenyl-4,4,5,5-tetramethylimidazoline-1-oxyl 3-oxide Radical (PTIO•) Trapping Activity and Mechanisms of 16 Phenolic Xanthones
Source: Molecules. 2018 Jul 11;23(7):1692. doi: 10.3390/molecules23071692 (PMC6100357; doi:10.3390/molecules23071692)
Supplement: Supplementary file 1 [file molecules-23-01692-s001.zip › Suppl/Suppl. 16 Appearance and analysis certificate of neomangiferin.pdf]

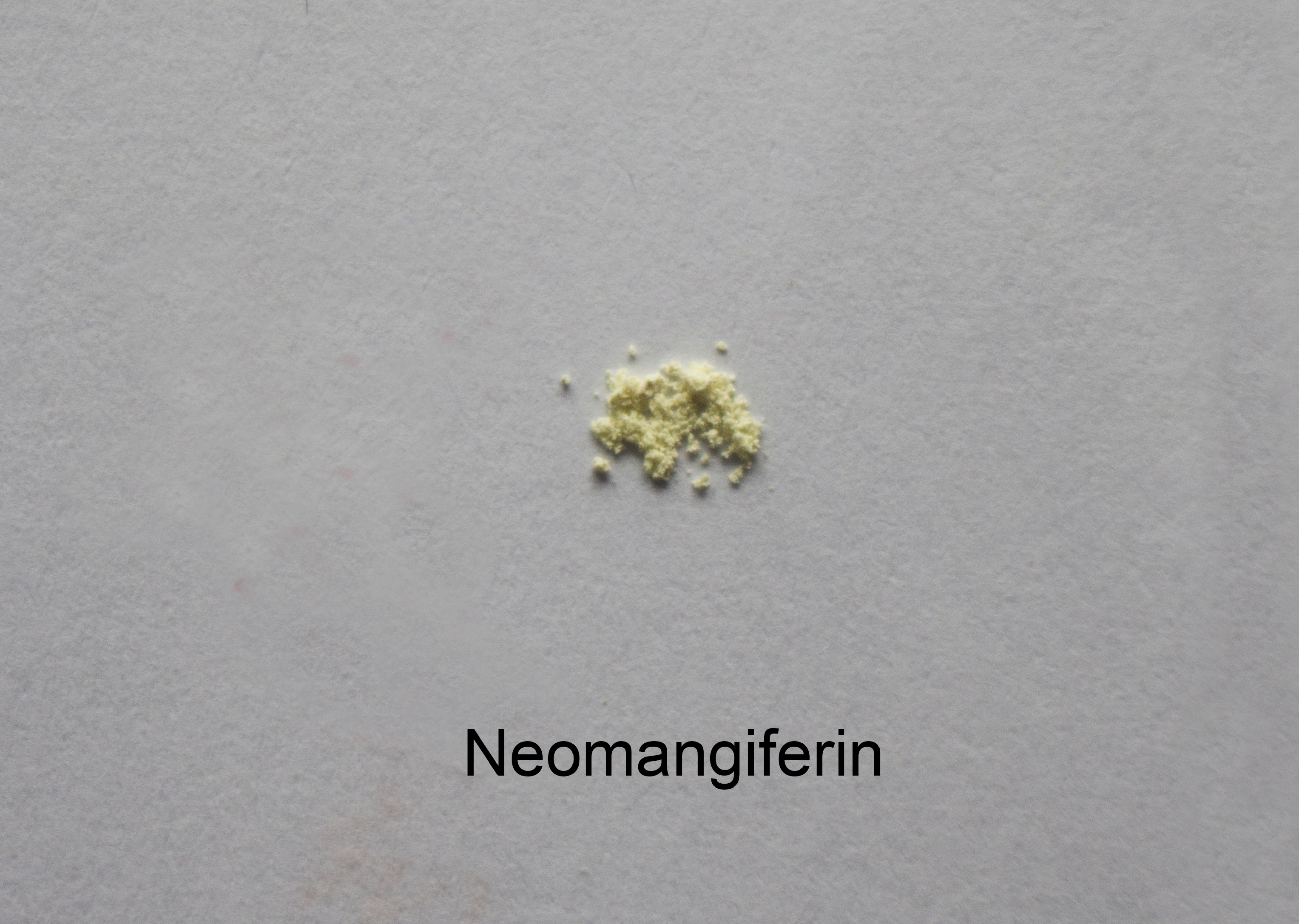

Neomangiferin

成都普瑞法科技开发有限公司
Chengdu Biopurify Phytochemicals Ltd.

文件编码（DOC NO.）：PRF-COA-BP099301

产品分析证书
Certificate of Analysis

中文名称：新芒果苷

English Name：Neomangiferin
别名(Alias):

产品编码(Cat. No.):BP0993
CAS Number: 64809-67-2
分子式(M. F.): C25H28O16
分子量(M. W.): 584.483

批号(Batch No.): PRF7080245
报告日期(Report date): 2016/8/2

Structure

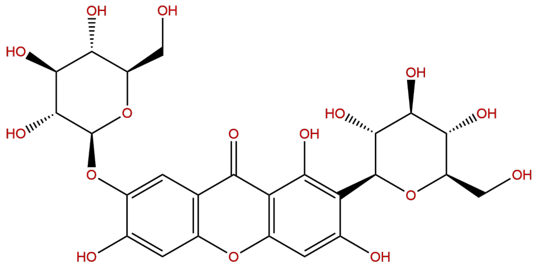

检验结果（Analytical result）：

检验项目（Test Item）	检验指标（Specifications）	检验结果（Results）
外观Appearance	Light yellow powder	Light yellow powder
干燥失重Loss on drying	＜3.0%	1.6%
纯度Purity (HPLC-DAD, 255nm)*	≥98.0%	99.01%
质谱Mass	584.483±1	Conforms
核磁NMR	Comply with the structure	Conforms

*** 色谱图见附件（Please find HPLC chromatography attached.）**

贮存条件（Storage）:类白色粉末
复测期（Retest date）:two years (2018-08-01) under conditions list above.
备注（Remarks）：如遇质量问题，请于收到产品之日起 15 日内与我们联系。
In case of quality issue, please contact us within 15 days after receipt of the product.

QC: Zhang Ling　　　　QA: Wu Qi

Date: 2016年8月2日　　　　Date: 2016年8月2日

Tel：+86-28-82633987　Fax：+86-28-82633165
http://www.biopurify.com　Email：sales@biopurify.com　biopurify@gmail.com

SAMPLE INFORMATION

Sample Name:	Neomangiferin PRF7080245	Acquired By:	System
Sample Type:	Unknown	Sample Set Name:	
Vial:	75	Acq. Method Set:	Neomangiferin
Injection #:	1	Processing Method:	Samples
Injection Volume:	5.00 ul	Channel Name:	255.0nm
Run Time:	25.0 Minutes	Proc. Chnl. Descr.:	PDA 255.0 nm
Date Acquired:	2016-8-2 16:42:17 CST		
Date Processed:	2016-8-2 9:44:24 CST		

Auto-Scaled Chromatogram

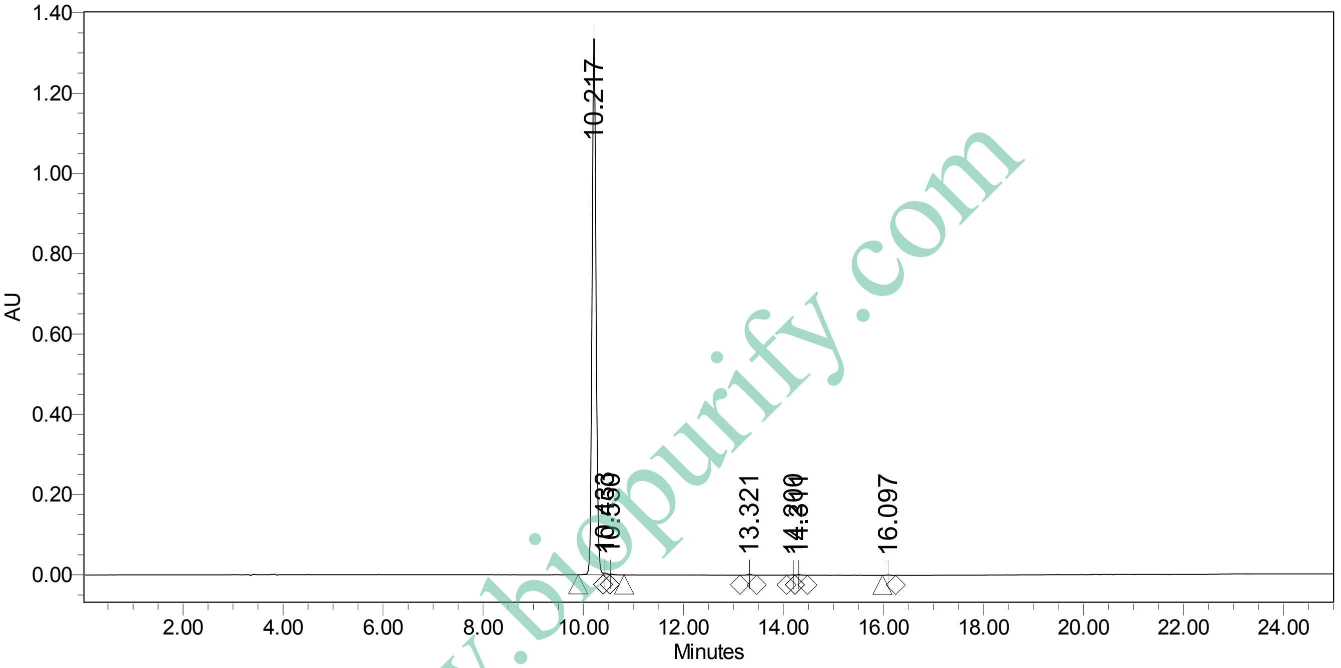

www.biopurify.com

Peak Results

	Name	RT	Area	% Area	USP Plate Count	USP Resolution
1		10.217	7293532	99.01	76268.34	
2		10.433	24893	0.34		1.47
3		10.550	9685	0.13		
4		13.321	15027	0.20	107962.87	
5		14.200	5541	0.08		5.53
6		14.311	12321	0.17	121672.54	0.61
7		16.097	5290	0.07	177997.19	10.11
