# Supplementary material for: 2-Phenyl-4,4,5,5-tetramethylimidazoline-1-oxyl 3-oxide Radical (PTIO•) Trapping Activity and Mechanisms of 16 Phenolic Xanthones
Source: Molecules. 2018 Jul 11;23(7):1692. doi: 10.3390/molecules23071692 (PMC6100357; doi:10.3390/molecules23071692)
Supplement: Supplementary file 1 [file molecules-23-01692-s001.zip › Suppl/Suppl. 17 Appearance and analysis certificate of lancerin.pdf]

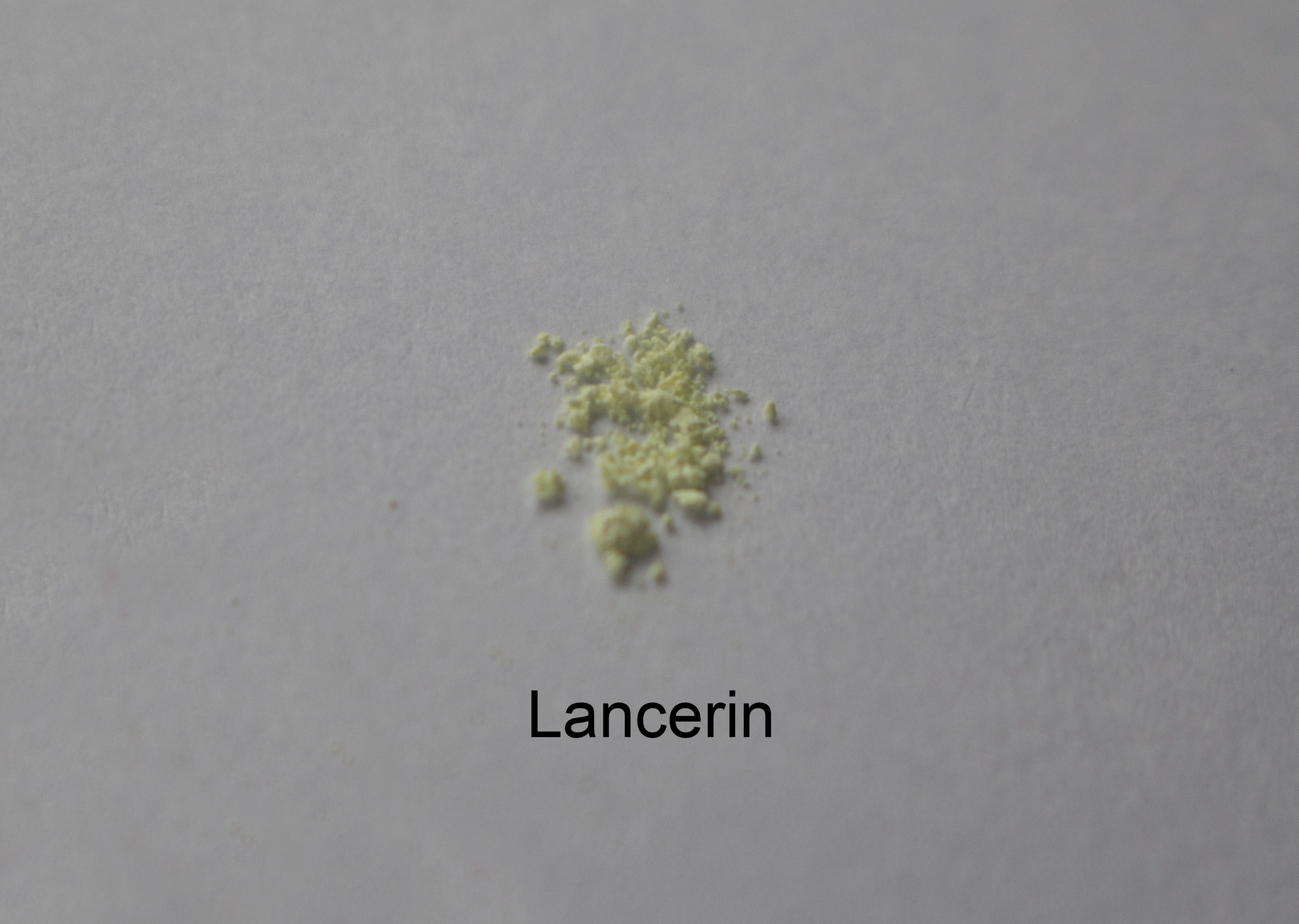
Lancerin

成都普瑞法科技开发有限公司
Chengdu Biopurify Phytochemicals Ltd.

Add: No.11 Building, No. 388 Rongtaidadao CNSTP
Wenjiang Zone, Chengdu, Sichuan, 611130 China
TEL: 028-82633987 FAX: 028-82633165
E-mail: biopurify@gmail.com sales@biopurify.com
Web: www.biopurify.com

Certificate of Analysis

Product Name: Lancerin

Other Name:

Catalogue No.: BP3227

Batch No.: PRF7061205

Reported Date: 2016-06-12

CAS Number: 81991-99-3

Mol. Formula: $C_{19}H_{18}O_{10}$

Mol. Weight: 406.343

Analysis Method of Purity: HPLC-DAD

Structure:

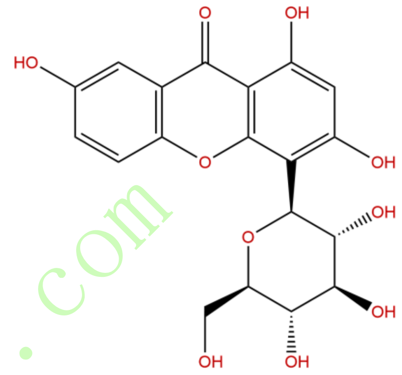

Analytical result:

**Test**	**Specification**	**Results**
Appearance	Pale yellow powder	Pale yellow powder
Loss on drying	＜3.0%	1.3%
Purity (HPLC, 320 nm)*	≥98.0%	99.19%

***** Please find HPLC chromatography attached.

Package: Brown vial or HDPE Plastic Bottle

Storage: Cool and Dry place, protected from light, keep package airproofed when not in use.

Expiration: two years (2018-06-12) under conditions list above**.**

QC: MengPan
Date: 2016-06-12

QA: Lianglei Zhang
Date: 2016-06-12

www.biopurify.com

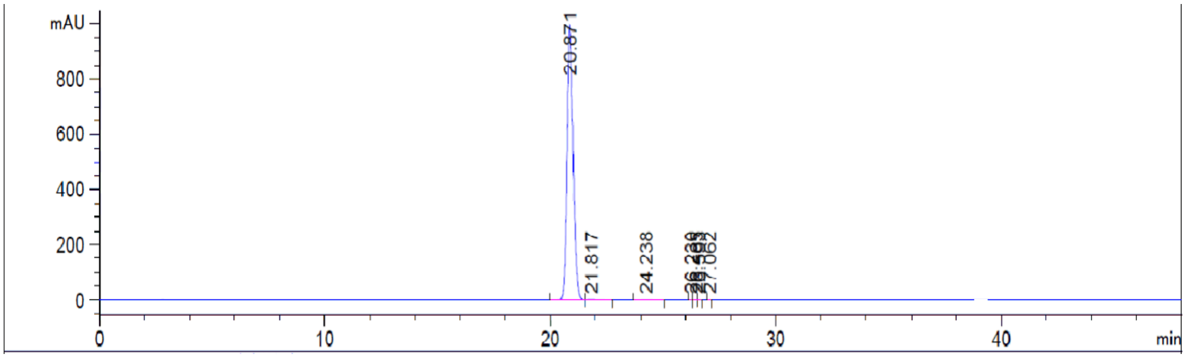

峰 #	保留时间 [min]	类型	峰宽 [min]	峰面积 [mAU*s]	峰高 [mAU]	峰面积 %
1	20.871	BV	0.2978	1.88864e4	993.37604	99.1953
2	21.817	VB	0.3330	109.13214	4.80417	0.5732
3	24.238	BB	0.2581	34.23557	1.83164	0.1798
4	26.230	BV	0.0741	1.16161	2.50561e-1	6.101e-3
5	26.485	VV	0.0811	4.36628	8.33443e-1	0.0229
6	26.593	VB	0.0698	2.22354	4.82017e-1	0.0117
7	27.062	BB	0.0729	2.08749	4.60427e-1	0.0110

www.biopurify.
