# Supplementary material for: 2-Phenyl-4,4,5,5-tetramethylimidazoline-1-oxyl 3-oxide Radical (PTIO•) Trapping Activity and Mechanisms of 16 Phenolic Xanthones
Source: Molecules. 2018 Jul 11;23(7):1692. doi: 10.3390/molecules23071692 (PMC6100357; doi:10.3390/molecules23071692)
Supplement: Supplementary file 1 [file molecules-23-01692-s001.zip › Suppl/Suppl. 18 Original MS spectra.docx]

**Supplemental Material-18 Original MS spectra**

2-Phenyl-4,4,5,5-tetramethylimidazoline-1-oxyl 3-oxide Radical (PTIO•) Trapping Activity and Mechanisms of 16 Phenolic Xanthones

Xican Li ^1, 2, †, *^, Ban Chen ^1, 2, †^, Xiaojun Zhao ^1, 2^, Dongfeng Chen ^3, 4, *^

^1^ School of Chinese Herbal Medicine, Guangzhou University of Chinese Medicine, Waihuan East Road No. 232, Guangzhou Higher Education Mega Center, Guangzhou 510006, China; imchenban@foxmail.com (B.C.)

^2^ Innovative Research & Development Laboratory of TCM, Guangzhou University of Chinese Medicine, Waihuan East Road No. 232, Guangzhou Higher Education Mega Center, Guangzhou 510006, China;

^3^ School of Basic Medical Science, Guangzhou University of Chinese Medicine, Waihuan East Road No. 232, Guangzhou Higher Education Mega Center, Guangzhou 510006, China;

^4^ The Research Center of Basic Integrative Medicine, Guangzhou University of Chinese Medicine, Waihuan East Road No. 232, Guangzhou Higher Education Mega Center, Guangzhou 510006, China;

***** Correspondence: lixican@126.com or lixc@gzucm.edu.cn (X.L.); [chen888@gzucm.edu.cn](mailto:chen888@gzucm.edu.cn) (D.C.);
Tel.: +86-203-935-8076 (X.L.)

**^†^** These authors contributed equally to this work.

Figure S1A. Chromatogram of possible dimeric products of garcinone C when the formula [C_46_H_50_O_14_-H]^-^ was extracted.

Figure S1B. Primary MS spectra of garcinone C - garcinone C dimer.

Figure S1C. Secondary MS spectra of garcinone C - garcinone C dimer.

Figure S2A. Chromatogram of possible dimeric products of γ-mangostin when the formula [C_46_H_46_O_12_-H]^-^ was extracted.

Figure S2B. Primary MS spectra of γ-mangostin-γ-mangostin dimer.

Figure S2C. Secondary MS spectra of γ-mangostin-γ-mangostin dimer.

Figure S3A. Chromatogram of possible dimeric products of Subelliptenone G when the formula [C_26_H_14_O_10_-H]^-^ was extracted.

Figure S3B. primary MS spectra of Subelliptenone G - Subelliptenone G dimer.

Figure S4A. Chromatogram of possible dimeric products of isomangiferin when the formula [C_38_H_34_O_22_-H]^-^ was extracted.

Figure S4B. Primary MS spectra of isomangiferin-isomangiferin dimer.

Figure S4C. Secondary MS spectra of isomangiferin-isomangiferin dimer.

Figure S5A. Chromatogram of possible dimeric products of 1,6,7-trihydroxyxanthone when the formula [C_26_H_14_O_10_-H]^-^ was extracted.

Figure S5B. Primary MS spectra of 1,6,7-trihydroxyxanthone -1,6,7-trihydroxyxanthone dimer.

Figure S5C. Secondary MS spectra of 1,6,7-trihydroxyxanthone -1,6,7-trihydroxyxanthone dimer.

Figure S6A. Chromatogram of possible dimeric products of 1,2,5-dihydroxyxanthone when the formula [C_26_H_14_O_10_-H]^-^ was extracted.

Figure S6B. Primary MS spectra of 1,2,5-dihydroxyxanthone - 1,2,5-dihydroxyxanthone dimer.

Figure S7A. Chromatogram of possible dimeric products of 1,5,6-trihydroxyxanthone when the formula [C_26_H_14_O_10_-H]^-^ was extracted.

Figure S7B. Primary MS spectra of 1,5,6-trihydroxyxanthone-1,5,6-trihydroxyxanthone dimer.

Figure S7C. Secondary MS spectra of 1,5,6-trihydroxyxanthone -1,5,6-trihydroxyxanthone dimer.

Figure S8A. Chromatogram of possible dimeric products of norathyriol when the formula [C_26_H_15_O_6_-H]^-^ was extracted.

Figure S8B. Primary MS spectra of norathyriol-norathyriol dimer.

Figure S8C. Secondary MS spectra of norathyriol-norathyriol dimer.

Figure S9A. Chromatogram of possible dimeric products of 1,3,5,6-tetrahydroxyxanthone when the formula [C_26_H_14_O_12_-H]^-^ was extracted.

Figure S9B. Primary MS spectra of 1,3,5,6-tetrahydroxyxanthone - 1,3,5,6-tetrahydroxyxanthone dimer.

Figure S9C. Secondary MS spectra of 1,3,5,6-tetrahydroxyxanthone - 1,3,5,6-tetrahydroxyxanthone dimer.

Figure S10A. Chromatogram of possible dimeric products of isojacareubin when the formula [C_26_H_14_O_12_-H]^-^ was extracted.

Figure S10B. Primary MS spectra of isojacareubin - isojacareubin dimer.

Figure S10C. Secondary MS spectra of isojacareubin - isojacareubin dimer.

Figure S11A. Chromatogram of possible dimeric products of 1,3,5,8-tetrahydroxyxanthone when the formula [C_26_H_14_O_12_-H]^-^ was extracted.

Figure S11B. Primary MS spectra of 1,3,5,8-tetrahydroxyxanthone - 1,3,5,8-tetrahydroxyxanthone dimer.

Figure S11C. Secondary MS spectra of 1,3,5,8-tetrahydroxyxanthone - 1,3,5,8-tetrahydroxyxanthone dimer.

Figure S12A. Chromatogram of possible dimeric products of isomangiferin when the formula [C_38_H_34_O_22_-H]^-^ was extracted.

Figure S12B. Primary MS spectra of isomangiferin-isomangiferin dimer.

Figure S12C. Secondary MS spectra of isomangiferin-isomangiferin dimer.

Figure S13A. Chromatogram of possible dimeric products of 2-hydroxyxanthone when the formula [C_26_H_14_O_6_-H]^-^ was extracted.

Figure S13B. Primary MS spectra of 2-dihydroxyxanthone -2-dihydroxyxanthone dimer.

Figure S14A. Chromatogram of possible dimeric products of 7-O-methylmangiferin when the formula [C_40_H_38_O_22_-H]^-^ was extracted.

Figure S14B. Primary MS spectra of 7-O-methylmangiferin -7-O-methylmangiferin dimer.

Figure S15A. Chromatogram of possible dimeric products of lancerin when the formula [C_38_H_34_O_20_-H]^-^ was extracted.

Figure S15B. Primary MS spectra of lancerin -lancerin dimer.
