# Supplementary material for: 2-Phenyl-4,4,5,5-tetramethylimidazoline-1-oxyl 3-oxide Radical (PTIO•) Trapping Activity and Mechanisms of 16 Phenolic Xanthones
Source: Molecules. 2018 Jul 11;23(7):1692. doi: 10.3390/molecules23071692 (PMC6100357; doi:10.3390/molecules23071692)
Supplement: Supplementary file 1 [file molecules-23-01692-s001.zip › Suppl/Suppl. 2 Appearance and analysis certificate of garcinone C.pdf]

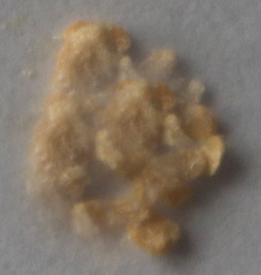

Garcinone C

成都普瑞法科技开发有限公司
Chengdu Biopurify Phytochemicals Ltd.

文件编码（DOC NO.）：PRF-COA-BP062201

产品分析证书
Certificate of Analysis

中文名称：1,3,6,7-四羟基-8-(3-羟基-3-甲基丁基)-2-(3-甲基-2-丁烯-1-基)-9H-占吨-9-酮

English Name: Garcinone C
别名(Alias):

产品编码(Cat. No.):BP0622
CAS Number: 76996-27-5
分子式(M. F.): C23H26O7
分子量(M. W.): 414.454

批号(Batch No.): PRF7082941
报告日期(Report date): 2016/8/30

Structure

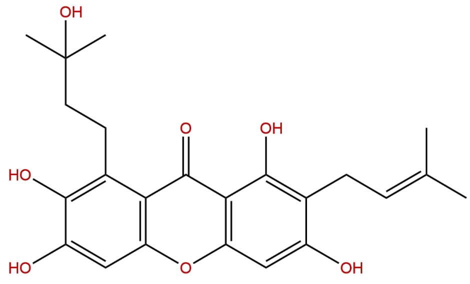

检验结果（Analytical result）：

检验项目（Test Item）	检验指标（Specifications）	检验结果（Results）
外观Appearance	Light yellow powder	Light yellow powder
干燥失重Loss on drying	＜3.0%	1.8 %
纯度Purity (HPLC-DAD,260nm)*	≥98.0%	99.37%
质谱Mass	516.5±1	Conforms
核磁NMR	Comply with the structure	Conforms

* 色谱图见附件（Please find HPLC chromatography attached.）

贮存条件（Storage）:2~8℃
复测期（Retest date）:two years (2018-08-29) under conditions list above.
备注（Remarks）：如遇质量问题，请于收到产品之日起 15 日内与我们联系。
In case of quality issue, please contact us within 15 days after receipt of the product.

QC: Zhang Ling　　　　QA: Wu Qi

Date: 2016年8月30日　　　　Date: 2016年8月30日

Tel：+86-28-82633987　Fax：+86-28-82633165
http://www.biopurify.com　Email：sales@biopurify.com　biopurify@gmail.com

Empower2 software

Chengdu Biopurify Phytochemicals Ltd.

SAMPLE INFORMATION

Sample Name:	Garcinone C PRF7082941	Acquired By:	System
Sample Type:	Unknown	Sample Set Name:	
Vial:	8	Acq. Method Set:	Garcinone C
Injection #:	1	Processing Method:	sample
Injection Volume:	10.00 ul	Channel Name:	260.0nm
Run Time:	25.0 Minutes	Proc. Chnl. Descr.:	PDA 260.0 nm
Date Acquired:	2016-8-30 9:45:01 CST		
Date Processed:	2016-8-30 10:18:55 CST		

Auto-Scaled Chromatogram

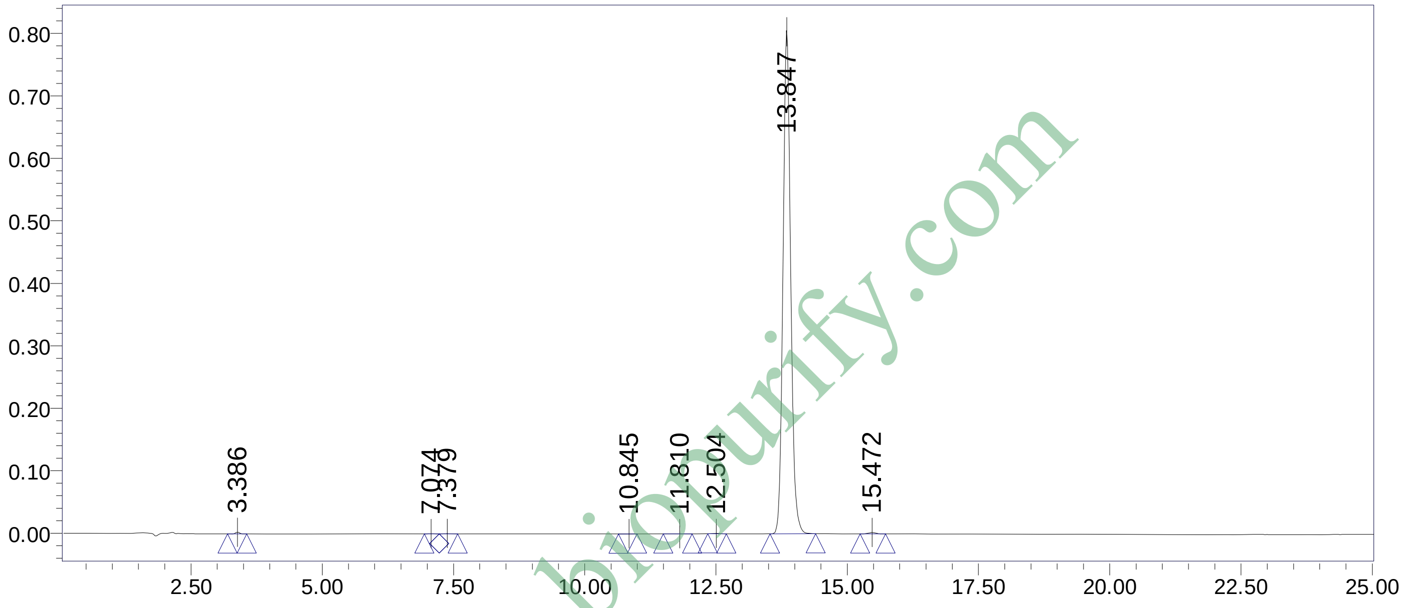

www.biopurify.com

Peak Results

	RT	Area	% Area	USP Plate Count	USP Resolution
1	3.386	14628	0.18	9423.50	
2	7.074	2027	0.02	22153.42	21.95
3	7.379	2780	0.03	22601.43	1.58
4	10.845	3679	0.04	33250.35	15.85
5	11.810	2603	0.03	19893.46	3.46
6	12.504	5233	0.06	36647.86	2.40
7	13.847	8169091	99.37	43920.63	5.04
8	15.472	21040	0.26	52958.57	5.96
