# Supplementary material for: 2-Phenyl-4,4,5,5-tetramethylimidazoline-1-oxyl 3-oxide Radical (PTIO•) Trapping Activity and Mechanisms of 16 Phenolic Xanthones
Source: Molecules. 2018 Jul 11;23(7):1692. doi: 10.3390/molecules23071692 (PMC6100357; doi:10.3390/molecules23071692)
Supplement: Supplementary file 1 [file molecules-23-01692-s001.zip › Suppl/Suppl. 3 Appearance and analysis certificate of a├-mangostin.pdf]

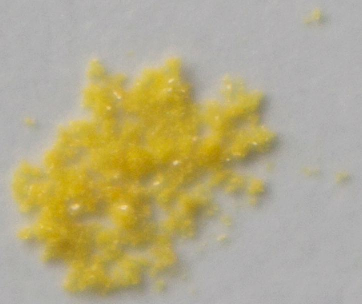

γ-Mangostin

文件编码(DOC No.): PRF-COA-BP061401

成都普瑞法科技开发有限公司
Chengdu Biopurify Phytochemicals Ltd.

产品分析证书

Certificate of Analysis

中文名：γ-倒捻子素

English Name： Gamma-mangostin

别名(Alias): Normangostin

Structure

产品图片

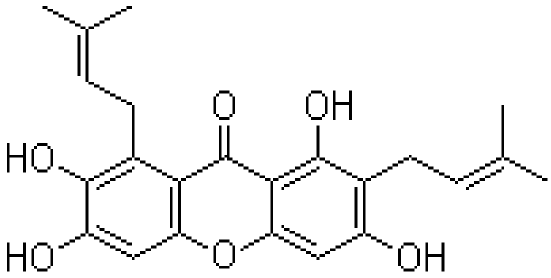

产品编码(Cat. No.): BP0614

CAS Number: 31271-07-5

分子式(M. F.): $C_{23}H_{24}O_6$

分子量(M. W.): 396.439

批号(Batch No.): PRF7012601

报告日期(Report date): 2016-01-26

检验结果（Analytical result）：

检验项目（Test Item）	检验指标（Specifications）	检验结果（Results）
外观Appearance	Yellow powder	Yellow powder
干燥失重Loss on drying	＜3.0%	0.36%
纯度Purity (HPLC-DAD, 317nm)*	≥98.0%	98.9%
质谱Mass	396.439 ±1.0	Conforms
核磁NMR	Comply with the structure	Conforms
结论（Conclusion）	合格（Qualified）	

* 色谱图见附件（Please find HPLC chromatography attached.）

包装（Package）: 棕色螺口玻璃瓶（Brown vial）

贮存条件（Storage）: 避光密闭保存于阴凉干燥处。长期保存建议-20℃。

Cool and Dry place, protected from light, keep package airproofed when not in use.

Please store in -20℃ for long term storage

复测期（Retest date）: two years (2018-01-25) under conditions list above.

备注（Remarks）： 如遇质量问题，请于收到产品之日起 15 日内与我们联系。
In case of quality issue, please contact us within 15 days after receipt of the product.

QC: Zhang Ling　　　　**QA:** Wu Qi

Date: 2016-01-26　　　　**Date: 2016-01-26**

Tel：+86-28-82633987　　Fax：+86-28-82633165
http://www.biopurify.com　　Email：sales@biopurify.com　　biopurify@gmail.com

Empower2 software

Chengdu Biopurify Phytochemicals Ltd.

SAMPLE INFORMATION

Sample Name:	γ Mangostin PRF7012601	Acquired By:	System
Sample Type:	Unknown	Sample Set Name:	
Vial:	57	Acq. Method Set:	γ Mangostin
Injection #:	1	Processing Method:	Samples
Injection Volume:	10.00 ul	Channel Name:	317.0nm
Run Time:	25.0 Minutes	Proc. Chnl. Descr.:	PDA 317.0 nm
Date Acquired:	2016-1-26 15:46:41 CST		
Date Processed:	2016-1-26 16:12:55 CST		

Auto-Scaled Chromatogram

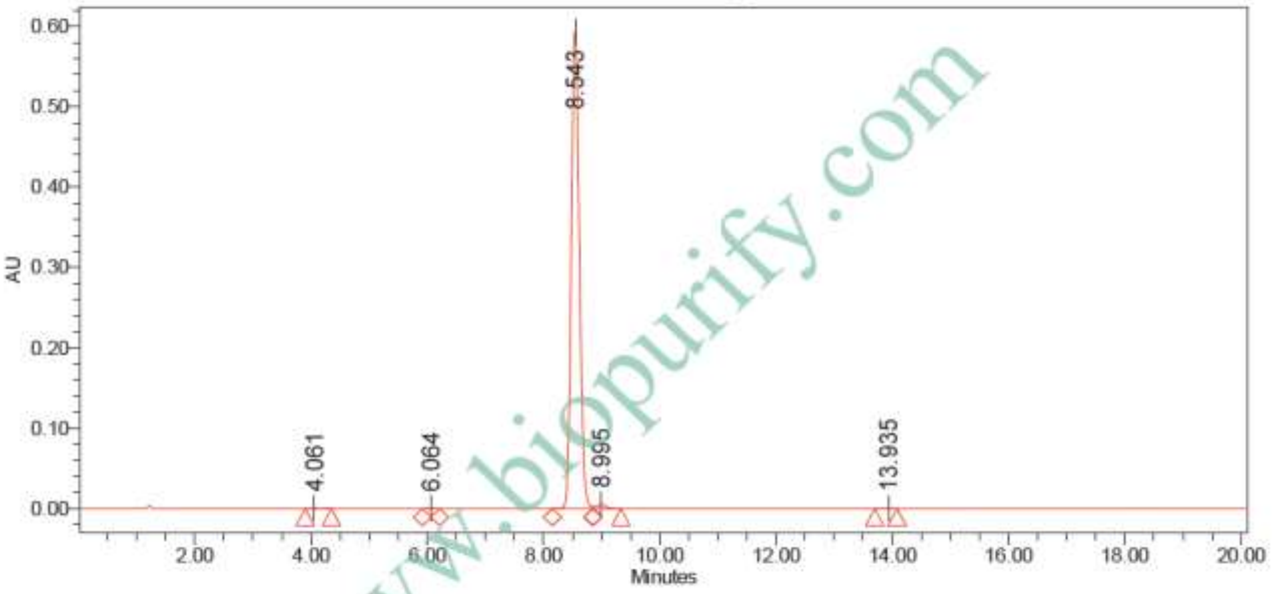

Peak Results

	Name	RT	Area	% Area	USP Plate Count	USP Resolution
1		4.061	2673	0.05	11714.49	
2		6.064	4545	0.08	10003.88	10.15
3		8.543	5700161	98.86	18194.41	9.93
4		8.995	55922	0.97	14324.21	1.55
5		13.935	2657	0.05	36258.55	16.57

Reported by User: System
Report Method: Chengdu
Report Method ID: 10505
Page: 1 of 1

Project Name: 2016
Date Printed:
2016-1-26
17:11:00 PRC

www.biopurify.com
