# Supplementary material for: 2-Phenyl-4,4,5,5-tetramethylimidazoline-1-oxyl 3-oxide Radical (PTIO•) Trapping Activity and Mechanisms of 16 Phenolic Xanthones
Source: Molecules. 2018 Jul 11;23(7):1692. doi: 10.3390/molecules23071692 (PMC6100357; doi:10.3390/molecules23071692)
Supplement: Supplementary file 1 [file molecules-23-01692-s001.zip › Suppl/Suppl. 5 Appearance and analysis certificate of mangiferin.pdf]

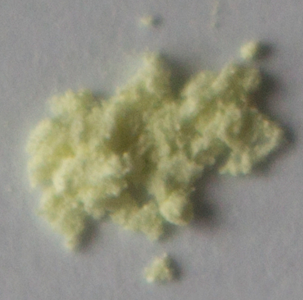

Mangiferin

文件编码（DOC NO.）：**PRF-COA-BP092201**

产品分析证书
Certificate of Analysis

中文名称：芒果苷

English Name：Mangiferin
别名(Alias): Euxanthogen; Chinomine; Alpizarin; Hedysaride; Mannipherin; Chedisaride; Shamimin

CAS Number: 4773-96-0
分子式(M. F.): C19H18O11
分子量(M. W.): 422.342

批号(Batch No.): PRF7090142
报告日期(Report date): 2016/9/1

Structure

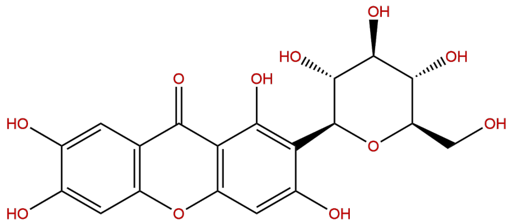

检验结果（Analytical result）：

**检验项目（Test Item）**	**检验指标（Specifications）**	**检验结果（Results）**
外观Appearance	Light yellow powder	Light yellow powder
干燥失重Loss on drying	＜3.0%	0.82 %
纯度Purity (HPLC-DAD, 257nm)*	≥98.0%	99.91%
质谱Mass	422.342±1	Conforms
核磁NMR	Comply with the structure	Conforms

*** 色谱图见附件（Please find HPLC chromatography attached.）**

贮存条件（Storage）:2~8℃
复测期（Retest date）:two years (2018-08-31) under conditions list above.
备注（Remarks）：如遇质量问题，请于收到产品之日起 15 日内与我们联系。
In case of quality issue, please contact us within 15 days after receipt of the product.

QC: Zhang Ling
Date: 2016年9月1日

QA: Wu Qi
Date: 2016年9月1日

SAMPLE INFORMATION

Sample Name:	Mangiferin PRF7090142	Acquired By:	System
Sample Type:	Unknown	Sample Set Name:	
Vial:	43	Acq. Method Set:	Mangiferin
Injection #:	1	Processing Method:	Samples
Injection Volume:	5.00 ul	Channel Name:	257.0nm
Run Time:	20.0 Minutes	Proc. Chnl. Descr.:	PDA 257.0 nm
Date Acquired:	2016-9-1 11:01:30 CST		
Date Processed:	2016-9-1 12:48:03 CST		

Auto-Scaled Chromatogram

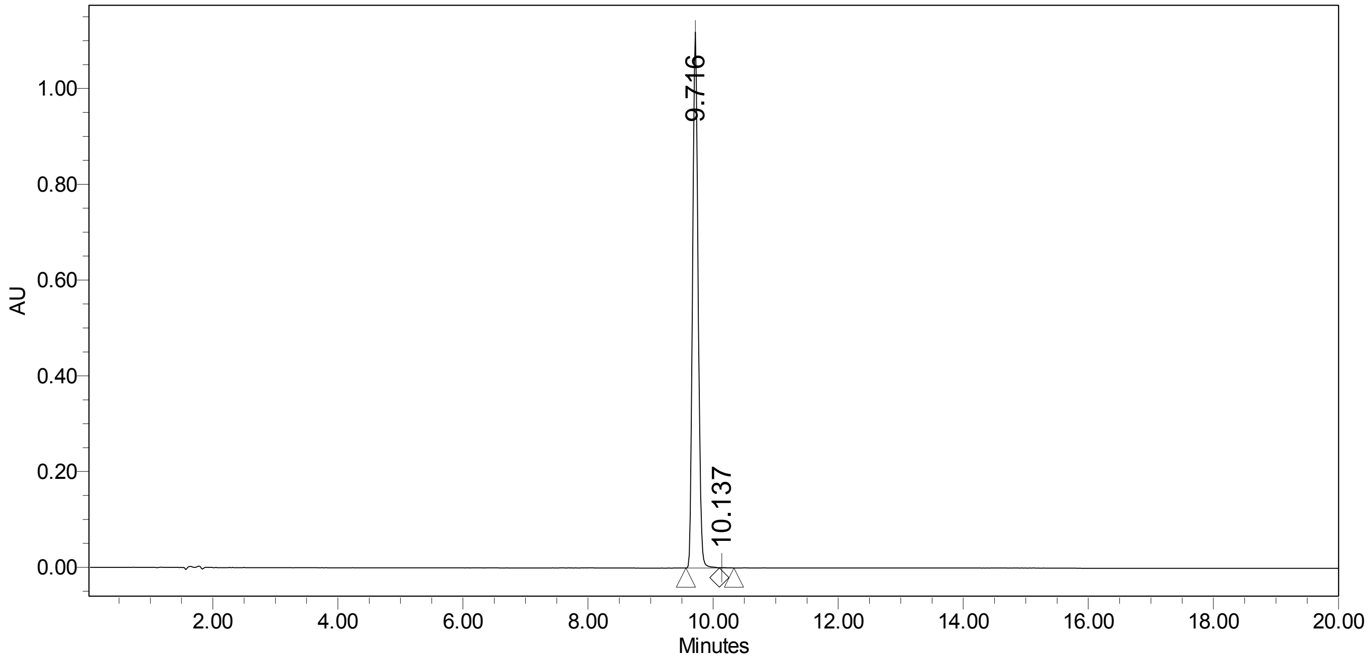

Peak Results

	Name	RT	Area	% Area	USP Plate Count	USP Resolution
1		9.716	6852966	99.91	55365.11	
2		10.137	5953	0.09		2.53
