# Supplementary material for: 2-Phenyl-4,4,5,5-tetramethylimidazoline-1-oxyl 3-oxide Radical (PTIO•) Trapping Activity and Mechanisms of 16 Phenolic Xanthones
Source: Molecules. 2018 Jul 11;23(7):1692. doi: 10.3390/molecules23071692 (PMC6100357; doi:10.3390/molecules23071692)
Supplement: Supplementary file 1 [file molecules-23-01692-s001.zip › Suppl/Suppl. 9 Appearance and analysis certificate of norathyriol.pdf]

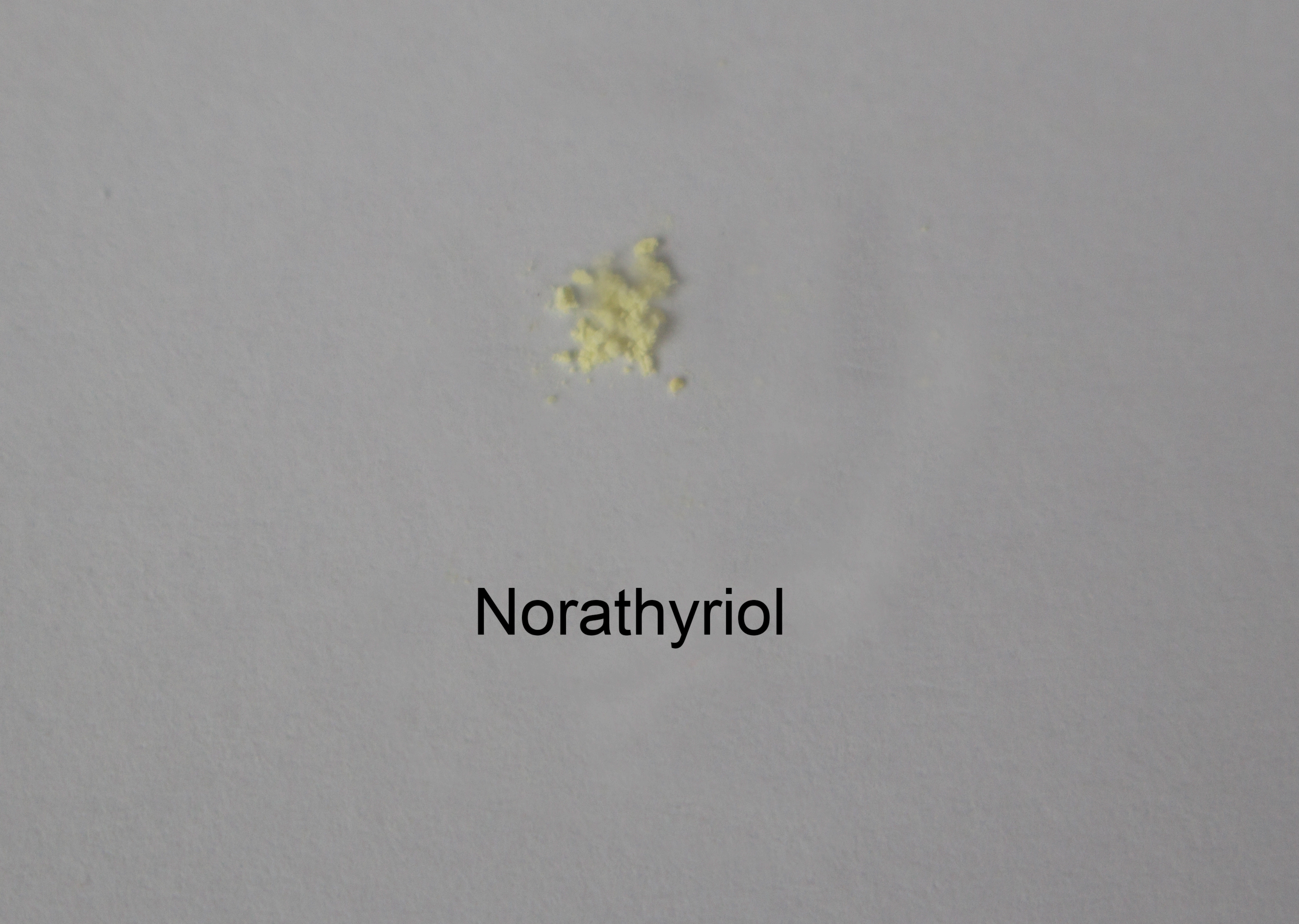
Norathyriol

西力生物
BBP BioBioPha

Natural Product Library for Drug Discovery

CERTIFICATE OF ANALYSIS

BBP No.: BBP02007

CAS No.: 3542-72-1

Chemical Name: Norathyriol

Molecular Formula: $C_{13}H_8O_6$

Structure:

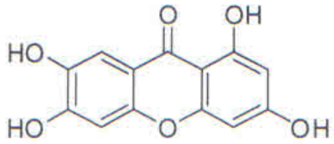

Purity: 98%

Appearance: Yellow powder

Solvent: Dimethyl sulfoxide, methanol

Exact Weight: 5.1 mg

Storage: Store in a dark place under the temperature of 0-4 °C

Intended Use: For laboratory use only

Reference: M. Hattori, et al., Phytochemistry, 1989, 28(4), 1289-1290

Warm Notice: When publishing, please cite as: ***chemical name*** was purchased from BioBioPha Co., Ltd. (Kunming, China)

Characterization Data Summary

Analytical Test	Results
Identification by ^{1}H-NMR	Consistent with the above structure
Purity tested by HPLC, ^{1}H-NMR	98%

Authorized Signature: Lina Zhang

Date: 2016-9-18

BioBioPha Co., Ltd.
2016.09.18
PASS

Tel.: +86 871 6521 7109 Fax: +86 871 6521 5563 E-mail: sales@mail.biobiopha.com

西力生物
BBP BioBioPha

Natural Product Library for Drug Discovery

PRODUCT QUALITY REPORT

Product Number: BBP02007

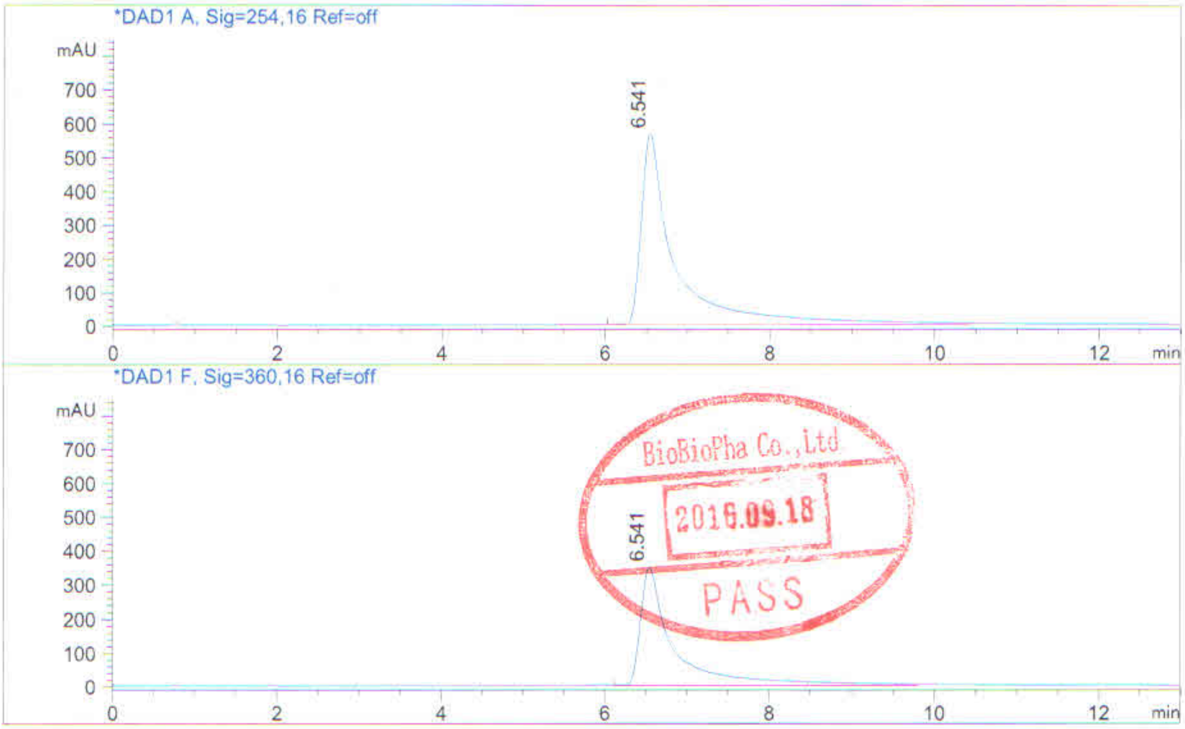

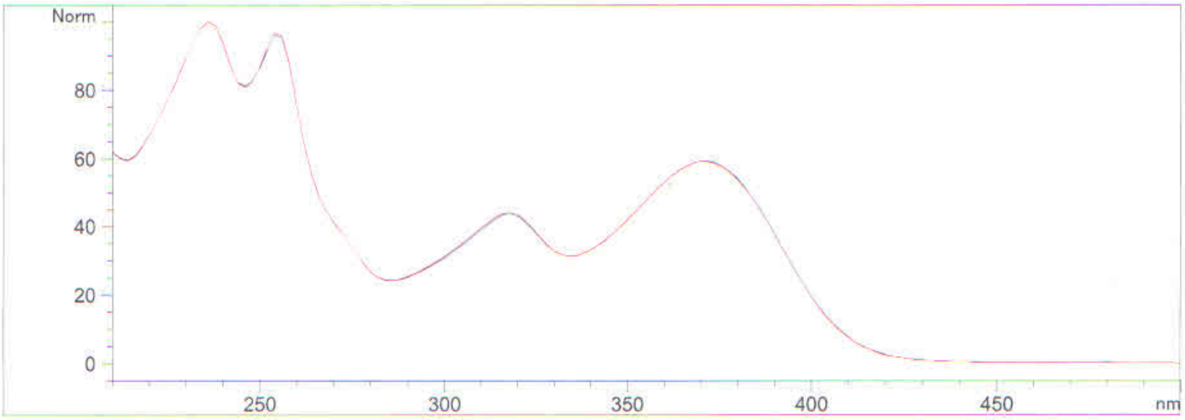

Agilent 1200 series HPLC system
Extend-C18 column (5 μm, 4.6 × 150 mm)
20%→100% MeOH in H_2O over 8.0 min followed by 100% MeOH to 13.0 min
1.0 ml/min, 20°C

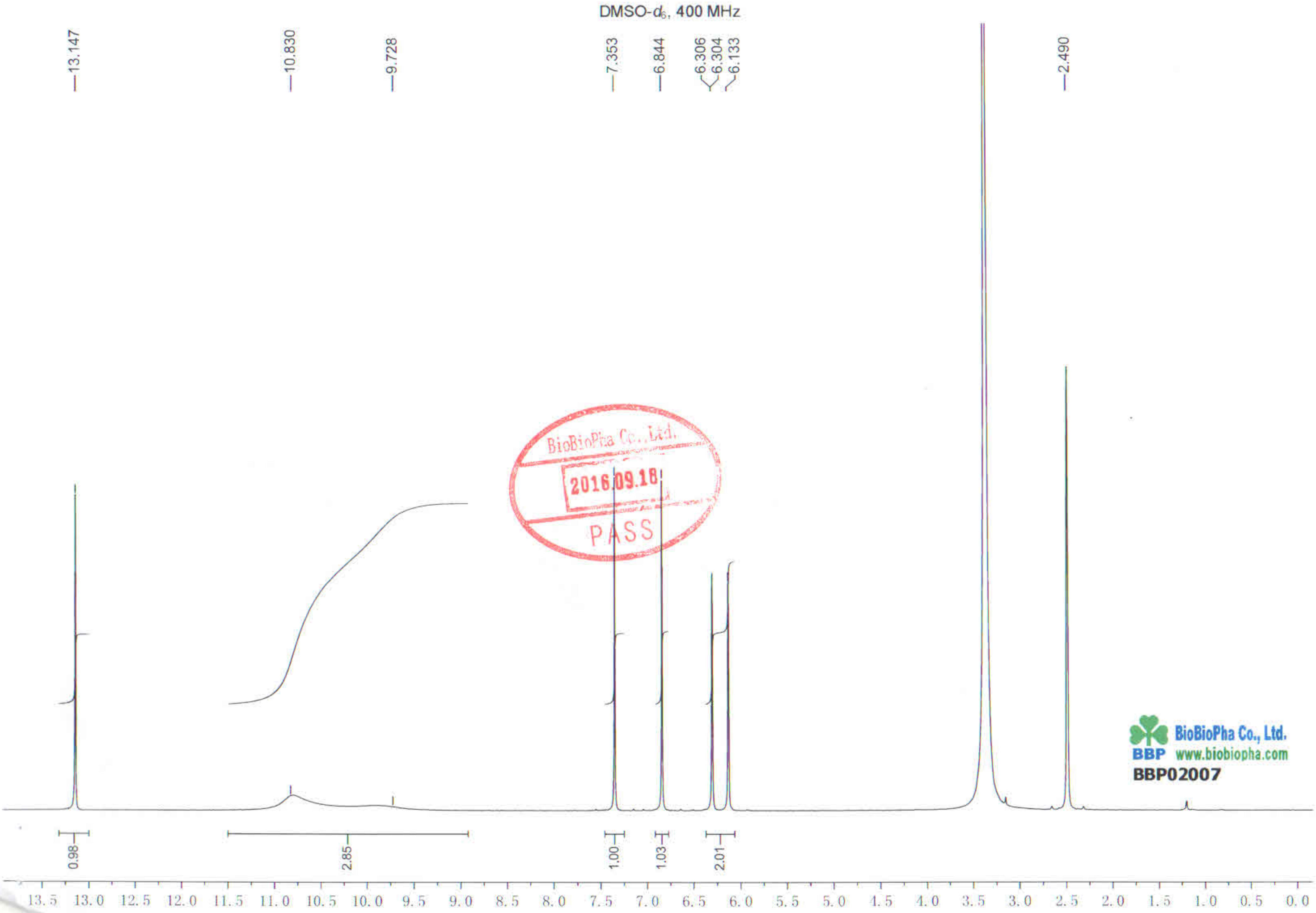
DMSO-d_6, 400 MHz
13.147
10.830
9.728
7.353
6.844
6.306
6.304
6.133
2.490
0.98
2.85
1.00
1.03
2.01
13.5 13.0 12.5 12.0 11.5 11.0 10.5 10.0 9.5 9.0 8.5 8.0 7.5 7.0 6.5 6.0 5.5 5.0 4.5 4.0 3.5 3.0 2.5 2.0 1.5 1.0 0.5 0.0
BioBioPha Co., Ltd.
2016.09.18
PASS
BioBioPha Co., Ltd.
BBP www.biobiopha.com
BBP02007
